# Perceptions and knowledge in primary health care and career intention at the first level of care: a cross-sectional study^*^

**DOI:** 10.1590/1518-8345.8021.4973

**Published:** 2026-10-23

**Authors:** Maricela Curisinche-Rojas, Tula Margarita Espinoza-Moreno, Reyna Ysmelia Peralta-Gómez, Lucy Zenelia Barrantes-Cruz, Gilmer Solis-Sánchez, Irma Yupanqui-Martinez, Lida Inés Carhuas-Peña, Cindy Vargas-Cabrera, Juvita Dina Soto-Hilario, Tomás Caycho-Rodriguez

**Affiliations:** 1 Universidad Científica del Sur, Facultad de Enfermería, Lima, Peru.; 2 Universidad Nacional Mayor de San Marcos, Lima, Peru.; 3 Universidad Nacional de San Agustín, Departamento Académico de Enfermería, Arequipa, Peru.; 4 Universidad Privada Antenor Orrego, Trujillo, La Libertad, Peru.; 5 Universidad Científica del Sur, Lima, Peru.; 6 Universidad Nacional Daniel Alcides Carrión, Facultad de Ciencias de la Salud-Escuela de Formación Profesional de Enfermería, Cerro de Pasco, Pasco, Peru.; 7 Universidad Nacional de Huancavelica, Facultad de Enfermería, Huancavelica, Peru.; 8 Universidad Señor de Sipán, Escuela de Enfermería, Chiclayo, Lambayeque, Peru.; 9 Universidad Nacional Hermilio Valdizán, Huánuco, Peru.

**Keywords:** Nursing, Primary Health Care, Perception, Knowledge, Intention, Work.

## Abstract

**Objective:**

to determine the association between perceptions of nursing work and knowledge of primary health care with regard to career intentions in primary care.

**Method:**

an analytical cross-sectional study was conducted with 740 university nursing students. Career intentions in primary care, perceptions of nursing work, and knowledge of primary health care were assessed using validated ad hoc instruments. A bivariate analysis and a median quantile regression analysis were performed.

**Results:**

a high career intention (66.8%), an unfavorable perception of nursing work (53.0%), and a moderate level of knowledge (67.0%) were predominant. Career intention was positively associated with perceptions of the work (adjusted coefficient: 0.46, 95% CI: 0.39-0.53), with self-perceived readiness for the job, and with plans to pursue a specialization. Male gender and the intention to study at private universities were negatively associated. No significant association was found with knowledge.

**Conclusion:**

favorable perceptions of nursing work in primary care increase the intention to pursue a career at this level, while knowledge about primary care does not appear to have an influence. Addressing perceptions of the work during university education is essential to promote the professional interest of future nurses.

## Highlights

**(1)**Future nurses in primary care will help reduce gaps in nursing.

**(2)**Favorable perception of nursing increases career intention in primary care.

**(3)**The average level of knowledge about primary health care was predominante.TT

**(4)**In private universities and among males, the intention to pursue a career in primary care is lower.

**(5)**The academy must build a positive view of nursing at the first level of care.

## Introduction

“Leaving no one behind” and achieving universal health coverage (UHC) worldwide are becoming increasingly distant goals. We face a troubling global landscape of stagnation in UHC, with more than 50% of the world’s population lacking full coverage for essential health services and many families facing catastrophic health expenditures^1^. In Latin American countries, there are still high rates of unmet health needs and various barriers to access that limit the performance of primary health care (PHC)^2^ ^-^ ^3^. It is now urgent to consolidate health systems with a focus on PHC as the central pillar of their organization and management. PHC is the most inclusive, pro-equity, cost-effective, and efficient strategy for overcoming barriers to access, increasing financial protection for families, and advancing toward the Sustainable Development Goals (SDGs)^1^ ^-^ ^2^. In Peru, efforts and progress to position PHC as the foundation of the health system are evident; however, moving from a conceptual model to authentic implementation still constitutes a slow process^4^.

First-level care (FLC) is essential for ensuring the effectiveness of PHC’s attributes (first point of contact, continuity, comprehensiveness, and coordination). It constitutes the gateway and foundation of the health pyramid, with a broad presence in rural and marginal urban communities, and is integrated and coordinated with other levels of care to ensure continuity of care. This level provides primary care (PC), which includes comprehensive, lifelong health care for individuals, families, and communities. In addition, it engages in health promotion and the prevention of risks and harm in partnership with organized communities and local governments to address the social determinants of health^5^. However, the FLC faces serious challenges that limit its performance^2^ ^,^ ^6^, with the shortage of nurses being a critical factor^7^ ^-^ ^8^. 

Nurses constitute the main workforce in healthcare and play a fundamental role in the FLC and PHC^8^, particularly in rural and less developed areas^9^. Given their broad professional profile and health-promoting approach, they are specialists in the comprehensive care of individuals, families, and communities^10^. The practice of nursing is the essence of PHC^7^, its leadership and autonomy in healthcare, and its social, ethical, and humanistic responsibility toward PHC are widely reported globally^7^ ^,^ ^11^ and in Latin America^9^ ^,^ ^12^. However, in Latin America, FLC nurses face unfavorable working conditions that limit the practice of PC and have a negative influence on job satisfaction and inclinations to remain in FLC^6^ ^,^ ^9^ ^,^ ^13^ ^-^ ^14^; similar to what has been observed in other regions^15^. Among these, shortages and unequal distribution of nurses, work and administrative overload, long shifts, scarce resources, inadequate infrastructure, work-related stress, insufficient recognition, and job insecurity, among others ^(^ ^6^ ^,^ ^9^ ^,^ ^13^ ^-^ ^14^ ^),^ are of note, and this situation is not foreign to Peruvian reality^16^. The absence or turnover of nurses in FLC affects access to PC^17^, and the attraction and retention of these professionals in FLC are fundamental. Indeed, evidence indicates that these are linked to optimal structural conditions in practice, favorable perceptions, and well-being at work^15^.

Students represent the future strategic reserve of nursing, which will help fill gaps in primary care. Therefore, it is a priority to be attentive to the career expectations and intentions of these students, as well as to explore the factors that facilitate or limit them. In Latin America, there are few precedents in this regard. Two studies in Mexico and Peru noted low expectations regarding work in primary care and a preference for hospital settings^18^ ^-^ ^19^; however, they did not conduct an in-depth analysis of the factors or explanations for this trend, which highlights the need for further research in this area. 

Evidence from other parts of the world indicates that nursing students have low preference for working in primary care settings^20^ ^-^ ^23^ and that inadequate perceptions of primary health care and the role of nursing-in terms of prestige, salary, and working conditions-act as barriers to choosing the FLC/PC as a professional field. In contrast, rural background, autonomy, and adequate curricular exposure are recognized as facilitating factors^20^ ^-^ ^23^. In Peru, it is unknown what is happening in this regard. 

In this context, this study is based on the principle that Peruvian nursing students nearing graduation or recent graduates who have favorable perceptions of the role of nursing in the PNA, as well as those who possess adequate knowledge of PHC acquired during their professional training, would have a stronger career intention in PHC. Accordingly, the research question was: Are perceptions of the nurse’s role in the PNA and knowledge of PHC associated with nursing students’ career intentions in the FLC at Peruvian universities? 

The objective of this study was to determine the association between nursing students’ perceptions of nursing work in the PNA and their knowledge of primary health care, in relation to their career intentions within the FLC in Peru. The evidence generated will support decision-making to improve curricula and the processes of training, planning, and aligning training offerings with the actual demand of the health care system, as well as motivation and incentive programs to attract and retain young talent in the FLC and policies to strengthen the FLC. 

## Method

### Study design 

We conducted an observational, cross-sectional, and analytical study, following the guidelines of *Strengthening the Reporting of Observational Studies in Epidemiology* (STROBE).

### Setting and data collection period

The study was conducted at 11 universities in Peru that offer nursing programs, both public and private, located in four major regions of the country: the north, the center, the south, and Metropolitan Lima. This distribution ensured the inclusion of representatives from regions with diverse social, educational, and health contexts.

The operational feasibility of the study (institutional approval and the availability of a co-researcher at the university) and the size of the student population were criteria used to select the universities. 

In the North, two universities located in coastal cities with high urban concentrations (Trujillo and Chiclayo) were included, with health services available in both urban and rural settings. In the central region, three universities in Andean areas (Huancavelica, Huánuco, and Cerro de Pasco) were included. These regions are characterized by predominantly rural and dispersed populations, higher poverty rates, and a high concentration of PNA health centers. In the South, one university was selected in Arequipa, the country’s second-largest city and a regional hub, offering highly specialized, top-tier services. Finally, in Metropolitan Lima-the country’s capital and the main academic and employment center in the health sector, characterized by its large student population and institutional diversity-five universities were chosen. 

Public and private universities were selected because both types reflect the diversity within higher education in nursing, with differences in academic training conditions and job market prospects depending on the type of institutional management. 

Data collection took place between November 2023 and February 2024.

### Population, selection criteria and sample

The study population consisted of nursing students enrolled in their third through fifth (final) years and graduates of the selected universities in 2022. Priority was given to students in advanced years due to their greater theoretical and practical exposure to PHC/FLC in the curriculum, and to recent graduates, to incorporate the perspective of the transition to the workforce.

The sample size was not estimated; all students who met the eligibility criteria, who were 18 years of age or older, and provided informed consent participated. Students who had temporarily withdrawn or were absent on the survey dates were excluded.

### Variables

The main variables were career intention to work in first-level care (IW-FLC), the nurses’ perception of work in first-level care (PNW-FLC) and knowledge of primary health care (K-PHC), which were measured numerically using Likert-type scale scores. PNW-FLC and IW-FLC were measured overall and for each dimension; furthermore, the distribution of scores across three levels was categorized into class intervals. 

Information on other covariates was collected based on the respondents’ characteristics: i) sociodemographic, ii) academic, and iii) occupational. At the sociodemographic level, the following were recorded: age, gender, marital status, have children (yes/no), and economic dependence; while the academic characteristics assessed were the type of university, the region of the country where the university was located, the year of study, whether they were considering a career change, previous education (at the technical or university level), the frequency of internships in FLC, preparation for work in FLC, specialization, master’s degree, and/or doctoral programs. Regarding employment, data were recorded on whether they had paid work, the sector, and workplace, and where they planned to work.

### Data collection instruments

PNW-FLC, IW-FLC, and K-PHC were measured using ad hoc scales developed specifically for this study. The scales for measuring PNW-FLC and IW-FLC consisted of statements or items with five Likert-type response options: Disagree, Partially agree, Moderately agree, Agree, Strongly agree, which were scored from 1 to 5, respectively. On the other hand, the K-PHC scale consisted of true-false statements, with a score of 0 for an incorrect answer and 1 for a correct one.

The proposed scale for the PNW-FLC consisted of 13 statements distributed across three dimensions: 1 - Image of nurses who acts in FLC, 2 - Work in FLC, and 3 - Economic consequences of working in FLC. On the other hand, the scale for measuring IW-FLC consisted of 10 statements distributed across three dimensions: 1 - Attitude toward working in FLC, 2 - Subjective normative beliefs, and 3 - Beliefs or self-perception of the ability to control behavior. The K-PHC scale had 31 statements, without distribution across dimensions. In addition, a specific questionnaire was used to assess sociodemographic, academic, and occupational characteristics. 

### Validation of data collection instruments

The development of the IW-FLC scale was reasoned in the Theory of Planned Behavior^24^ ^-^ ^25^, and the items for each dimension were formulated based on a review of the literature. Regarding the K-PHC scale, statements on the values, principles, and elements of PHC and the importance of a PHC-based health system were considered, identified through a review of scientific literature and technical documents. On the other hand, the PNW-FLC scale resulted from the restructuring and adaptation of a scale used with medical students in Latin America^26^ for the nursing context.

The three scales were validated by 11 expert judges, consisting of specialist nurses with master’s and/or doctoral degrees in public, community, and/or family health; In addition, they had more than 10 years of experience in the FLC and/or in the management of health programs at the regional or national level; they came from Lima and the regions involved in the study.

Using a shared online form, the raters developed validation matrices for the items on each scale, considering criteria of relevance, clarity and pertinency. The results were analyzed using Aiken´s V coefficient, yielding acceptable levels of agreement across all statements (items) (between 0.75 and 1.00 for PNW-FLC, between 0.73 and 0.97 for IW-FLC, and between 0.73 and 1.00 for K-PHC). 

The reliability of the instruments was determined based on internal consistency using McDonald’s Omega coefficient; it was found that, for each dimension of PNW-FLC, it was 0.908 (D1), 0.827 (D2) and 0.834 (D3); while for the IW-FLC dimensions they were 0.855 (D1), 0.914 (D2) and 0.880 (D3); in turn, for C-APS, this coefficient was 0.794.

Given the distribution of the PNW-FLC and IW-FLC items across dimensions, construct validity was assessed using confirmatory factor analysis, yielding acceptable fit indices for both scales (p<0.001). Specifically, for the PNW-FLC, a Comparative Fit Index (CFI) of 0.963, a Root Mean Square Error of Approximation *(*RMSEA) of 0.080 (95% confidence interval [95% CI]: 0.070; 0.102), and a Standardized Root Mean Square Residual *(*SRMR) of 0.03 were obtained. As for the IW-FLC scale, the CFI was 0.950, the RMSEA was 0.117 (95% CI: 0.103; 0.131), and the SRMR was 0.03.

### Data collection

Before data collection began, in-person and virtual meetings were held to coordinate and organize the study’s activities. Similarly, through virtual sessions, those responsible for data collection were trained in the procedures for obtaining informed consent and administering the instruments.

Data collection was conducted by faculty co-researchers from each university, who managed authorizations, coordinated dates, and recruited participants in institutional settings. After verifying eligibility criteria, the questionnaires were administered anonymously, without recording first or last names.

For graduates and 2022 students who did not attend the in-person meetings, a digital version of the instruments was used via Google Forms and shared via email or WhatsApp, with follow-up reminders to maximize the response rate.

### Data processing and analysis 

The collected data were organized in an MS Excel spreadsheet, and then analyzed by means of descriptive and inferential statistics, using the STATA v. 17.0 programs (Stata Corporation, College Station, Texas, USA), which made it possible to estimate the measures of absolute (n) and relative (%) frequency of the qualitative variables collected. The absolute and relative distribution of the frequency of answers to each question of the three (03) questionnaires applied was identified.

The scores obtained by summing each dimension and the total scale for each questionnaire were analyzed using measures of central tendency (mean and median) and dispersion (range, standard deviation, interquartile range [IQR]). Based on the scores for each scale, three class intervals were generated using Sturges’ rule, categorized as Unfavorable, Unfavorable, and Favorable for PNW-FLC; while for the IW-FLC and K-PHC, they were Low, Moderate, and High. These intervals were determined both overall and for each dimension of the scale. 

We assessed the difference in scores on the career intention questionnaire for each of its categories and on the total scale according to the categories of class intervals, the dimensions, and the total scale of the questionnaires on knowledge of PHC and nurses’ perceptions of their work in FLC, as well as according to the study categories and covariates. This comparison was performed using the Student’s t-test for independent samples or the Mann-Whitney U test, and one-way analysis of variance (ANOVA) or the Kruskal-Wallis H test. The choice of tests was based on the identification of normal or non-normal distribution (Shapiro-Wilk test) and the number of groups to be compared (2 or more than 3). 

The relationships between the career intention questionnaire score and the PHC knowledge questionnaire scores and nurses’ perceptions of their work in FLC were assessed using Pearson’s or Spearman’s correlation coefficients, depending on whether the data followed a normal or non-normal distribution (Shapiro-Wilk test). To assess the factors associated with the career intention questionnaire score, an adjusted median quantile regression was performed, considering as adjustment variables the independent variables (scores on the primary care knowledge questionnaires and perception of nurses’ work in FLC) and the covariates that had a p-value less than 0.20 in the crude model; Robust variances were used to avoid model bias caused by heteroscedasticity. All results were analyzed at a significance level of 0.05.

### Ethical considerations

Prior to the beginning of the study, the research protocol was approved by the Institutional Research Ethics Committee of the Universidad Científica del Sur (Certificate No. 055-CIEI-Científica-2023), and authorization obtained from the participating universities. Before administering the instruments, detailed information about the study was provided, and signed informed consent was obtained from students who agreed to participate. Related to the case of online data collection, the digital instrument (Google Forms) included a first section with a detailed explanation of the study, covering aspects related to student participation, and an electronic consent form in which they had to choose whether to participate. Students who agreed to participate completed the instruments voluntarily.

Personal data were protected through anonymization mechanisms, accessible only to researchers.

## Results

Of the 740 nursing students who participated in the study, the mean age was 24.4 ± 4.9 years; 85% of the participants were women, 89.2% were not married, 84.2% were childless, 74.3% were financially dependent, 76.4% were without paid employment, 59.5% were studying at public universities, 44.2% were studying at universities in Lima, 44.5% were in their fourth year of study, 71.9% did not plan to change careers, and 80.0% had no prior education. In terms of training, 32.0% had completed internships with moderate frequency, 44.3% reported being prepared to work in FLC, 64.3% indicated career intention in a “hospital, clinic, or specialized institute”, 87.4% planned to pursue specialized studies, 77.4% planned to pursue a master’s degree, and 53.6% planned to pursue a doctoral degree (Table 1).

**Table 1:** Characteristics of nursing students surveyed in Peru, 2023-2024 (n = 740)

Characteristics	n | Mean	% | SD*
Sociodemographic characteristics		
Age (Mean ± SD*)	24.4	4.9
Gender		
Female	629	85.0
Male	111	15.0
Marital status		
Single	660	89.2
Married	31	4.2
In a common-law marriage	41	5.5
Divorced	7	0.9
Other	1	0.1
Have children		
No	623	84.2
Yes	117	15.8
Economic dependence		
No	190	25.7
Yes	550	74.3
Academic aspects		
Type of university		
Public	440	59.5
Private	300	40.5
Region of the country where the university is located		
Central	178	24.1
Northern	106	14.3
Southern	129	17.4
Lima	327	44.2
Grade level:		
Third grade	80	10.8
Fourth grade	329	44.5
Fifth grade	264	35.7
Alumnus (former student)	67	9.1
Have you thought about changing careers?		
No	532	71.9
Yes	208	28.1
Previous education		
No	592	80.0
Yes	148	20.0
At the technical level	119	80.4
At the university level	29	19.6
Frequency of practices in the FLC^†^		
None or very few practices	103	13.9
A few practices	149	20.1
Moderately frequent practices	237	32.0
Frequent practices	174	23.5
Very frequent practices	77	10.4
Preparation for work on the FLC^†^		
I am not prepared	12	1.6
Somewhat prepared	102	13.8
Fairly prepared	256	34.6
I am prepared	328	44.3
I am very prepared	42	5.7
Do you have a plan to pursue specialized studies?		
No	13	1.8
I haven’t decided yet	80	10.8
Yes	647	87.4
Do you have plans to pursue a master’s degree?		
No	21	2.8
I haven’t decided yet	146	19.7
Yes	573	77.4
Do you plan to pursue a Ph.D.?		
No	35	4.7
I haven’t decided yet	308	41.6
Yes	397	53.6
Employment status		
Do you have a paid job?		
No	565	76.4
Yes	175	23.6
Sector where you work (n=175)		
Works in other sectors	67	38.3
Works in the health sector	108	61.7
Paid workplace (n=175)		
FLC^†^ health center or clinic	27	15.4
Hospital, clinic, or specialized institute	81	46.3
Other sectors not related to health care	67	38.3
Where do you intend to work?		
Hospital, clinic, specialized institute	478	64.6
FLC^†^ health center or clinic	153	20.7
Public or private research institutions	31	4.2
Non-clinical health-related organizations (Ministry of Health, PAHO^‡^, NGOs^§^, etc.)	40	5.4
Work as a professor at the university or technical college level	23	3.1
Other	15	2.0

*SD = Standard Deviation; ^†^FLC= First-level care; ^‡^PAHO = Pan American Health Organization; ^§^NGOs = Non-Governmental Organizations

According to the responses obtained from the questionnaires, the median score for IW-FLC and PNW-FLC assessments was 39 (IQR: 30 - 39), while the median score for K-PHC was 18 (IQR: 16-18). The scores were classified according to level, finding that 66.8% had a high assessment of IW-FLC, 53.0% had an unfavorable perception of the nurse’s work in FLC, and 67.0% of the students achieved a moderate level of knowledge about PHC (Table 2).

The relationship between IW-FLC score and PNW-FLC showed a statistically significant correlation (p<0.001) with a Rho value of 0.531; while the relationship between IW-FLC score and K-PHC showed a statistically significant inverse correlation with a Rho value of -0.166 (Table 3).

IW-FLC scores varied according to the categories of certain variables (p<0.05), with higher scores found among female students, students with economic dependency, attending a public university, in their third year, and not considering a career change, indicating that internships in the FLC were very frequent and that they consider themselves well-prepared to work in the FLC. Regarding future study intentions, both those intending to pursue a specialization or master’s degree had higher scores for the IW-FLC. In relation to the intention to pursue doctoral studies, the highest scores were among those who had not yet decided to pursue such studies (Table 4).

**Table 2:** Scores and levels obtained in the IW-FLC*, PNW-FLC^†^, and K-PHC^‡^ assessments among nursing students in Peru, 2023-2024 (n = 740)

Variables	Scores			Level n (%)		
	Mean ± SD^§^	Median (IQR^∥^)	Range^¶^	Low/Unfavorable**	Moderate/Less Favorable**	High/Favorable**
IW-FLC*						
Dimension: Attitude toward the career in the FLC^††^	14.3 ±3.7	16 (12 - 16)	4 - 20	99 (13.4)	206 (27.8)	435 (58.8)
Dimension: Subjective normative beliefs (social pressure)	11.5 ±3.2	12 (9 - 12)	3 - 15	109 (14.7)	188 (25.4)	443 (59.9)
Dimension: Beliefs or self-perception regarding the ability to control behavior	11.2 ±3.1	12 (9 - 12)	3 - 15	105 (14.2)	239 (32.3)	396 (53.5)
Overall assessment of the IW-FLC*	36.9 ±9.3	39 (30 - 39)	10 - 50	28 (3.8)	218 (29.5)	494 (66.8)
IW-FLC*						
Dimension: Image of the nurse working in the FLC^††^	14.4 ±5.1	15 (10 - 15)	5 - 25	250 (33.8)	276 (37.3)	214 (28.9)
Dimension: Work in the FLC^††^	19.0 ±5.3	20 (16 - 20)	6 - 30	113 (15.3)	327 (44.2)	300 (40.5)
Dimension: Economic consequences related to work in the FLC^††^	5.5 ±2.0	6 (4 - 6)	2 - 10	551 (74.5)	144 (19.5)	45 (6.1)
Overall evaluation of the PNW-FLC^†^	38.9 ±11.3	39 (30 - 39)	13 - 65	76 (10.3)	392 (53.0)	272 (36.7)
K-PHC^‡^						
Overall evaluation of the K-PHC^‡^	19.0 ±3.8	18 (16 - 18)	10 - 30	0 (0.0)	496 (67.0)	244 (33.0)

*IW-FLC= Intention to career in first-level care; ^†^PNW-FLC= Nurses’ perception of work in first-level care; K-PHC^‡^= Knowledge of primary health care; ^§^SD = Standard Deviation; ^∥^IQR = Interquartile Range; ^¶^Range = Minimum and maximum values; **Value applicable to PNW-FLC; ^††^FLC = First-level care

**Table 3:** Assessment of the correlation between IW-FLC* scores with PNW-FLC^†^ and K-PHC^‡^ scores among nursing students in Peru, 2023-2024 (n = 740)

	Rho^§^	P-value
PNW-FLC^†^		
Dimension: Image of a nurse working in the FLC^∥^	0.508	<0.001
Dimension: Work in the FLC^∥^	0.485	<0.001
Dimension: Economic consequences related to work in the FLC^∥^	0.408	<0.001
Overall evaluation of the PNW-FLC^†^	0.531	<0.001
K-PHC^‡^		
Evaluation of the K-PHC^‡^	-0.166	<0.001

*IW-FLC= Career intention in first-level care; ^†^PNW-FLC= Nurses’ perception of work in first-level care; K-PHC^‡^ = Knowledge of primary health care; ^§^Rho = Spearman’s Rho coefficient; ^∥^FLC = First-level care

**Table 4:** IW-FLC* scores according to characteristics of nursing students in Peru during the 2023-2024 (n = 740)

Characteristics	IW-FLC* score			P-value
	Mean ± SD^§^	Median (IQR^‡^)	Range^§^	
Socio-demographic data				
Gender				
Female	37.3 ±9.1	40 (31; 46)	10 - 50	0.028^∥^
Male	34.7 ±10.4	38 (27; 43)	10 - 50	
Marital status				
Single	37.2 ±9.0	39 (31; 45)	10 - 50	0.067^¶^
Married	37.8 ±11.0	40 (27; 47)	10 - 50	
In a common-law marriage	33.0 ±12.3	33 (21; 45)	10 - 50	
Divorced	29.3 ±10.5	27 (26; 30)	15 - 50	
Other	3.00 ±0.0	30 (30; 30)	30 - 30	
Have children				
No	37.2 ±9.0	40 (31; 45)	10 - 50	0.148^∥^
Yes	35.3 ±10.7	39 (26; 45)	10 - 50	
Financial dependence				
No	35.0 ±10.0	37.5 (27; 43)	10 - 50	0.002^∥^
Yes	37.6 ±9.0	40 (32; 46)	10 - 50	
Academic aspects				
Type of university		-		
Public	37.8 ±8.9	40 (32; 46)	10 - 50	0.003^∥^
Private	35.7 ±9.8	39 (28; 43)	10 - 50	
Grade level				
Third	40.0 ±9.0	45.5 (36; 46)	14 - 50	0.001^¶^
Fourth	36.5 ±9.5	39 (30; 44)	10 - 50	
Fifth	37.0 ±8.9	39 (31.5; 44)	10 - 50	
Alumnus (former student)	35.3 ±9.7	37 (28; 43)	10 - 50	
Have you considered changing careers?				
No:	37.7 ±9.3	40 (31; 46)	10 - 50	<0.001^∥^
Yes	35.1 ±9.1	37 (28; 41)	10 - 50	
Previous education				
No	37.3 ±9.1	40 (31; 46)	10 - 50	0.096^∥^
Yes	35.6 ±10.1	38 (27.5; 44)	10 - 50	
Completed previous education				
No	37.3 ±9.1	40 (31; 46)	10 - 50	0.249^¶^
Technical level	35.5 ±10.2	38 (27; 44)	10 - 50	
College level	35.7 ±9.7	39 (30; 41)	10 - 50	
Frequency of practices in FLC**				
None or very few	36.6 ±9.4	39 (30; 45)	10 - 50	<0.001^¶^
Few practices	35.0 ±8.9	38 (28; 40)	13 - 50	
Moderately frequent practices	38.0 ±9.5	40 (32; 46)	10 - 50	
Frequent practices	36.4 ±9.1	39 (30; 42)	10 - 50	
Very frequent practices	39.0 ±9.4	41 (34; 46)	14 - 50	
Preparation for work in FLC**				
I’m not ready	31.1 ±12.0	33 (24.5; 40)	10 - 49	<0.001^¶^
Somewhat ready	33.4 ±9.5	35 (25; 40)	13 - 50	
Mostly ready	35.7 ±8.9	38 (30; 42)	10 - 50	
I’m ready	38.8 ±8.9	40 (34; 46)	10 - 50	
I’m very ready	40.5 ±9.9	44 (33; 49)	20 - 50	
Do you plan to pursue specialized studies?				
No	26.5 ±12.5	24 (20; 36)	10 - 48	<0.001^¶^
I haven’t decided yet	33.8 ±9.2	35 (26; 40)	10 - 50	
Yes	37.5 ±9.1	40 (31; 46)	10 - 50	
Do you plan to pursue a master’s degree?				
No	30.8 ±12.3	31 (22; 41)	10 - 48	0.009^¶^
I haven’t decided yet	36.1 ±8.8	38.5 (29; 42)	13 - 50	
Yes	37.4 ±9.3	40 (31; 46)	10 - 50	
Do you plan to pursue a doctorate?				
No	30.2 ±10.8	31 (23; 39)	10 - 47	<0.001^¶^
I haven’t decided yet	37.9 ±9.3	40 (31; 46)	13 - 50	
Yes	36.8 ±9.0	39 (30; 43)	10 - 50	
Employment status				
Do you have a paid job?				
No	37.4 ±9.0	40 (31; 46)	10 - 50	0.151^∥^
Yes	35.3 ±10.2	37 (28; 43)	10 - 50	
Industry in which they work (n=175)				
Works in other sectors	36.7 ±9.5	39 (30; 45)	10 - 50	0.151^∥^
Works in the healthcare sector	34.4 ±10.5	35 (26; 42)	10 - 50	
Where do you plan to work?				
Hospital, clinic, specialized institute	36.3 ±9.3	39 (30; 44)	10 - 50	<0.001^¶^
FLC** health center or clinic	40.3 ±8.0	41 (38; 46)	13 - 50	
Public or private research institutions	38.6 ±8.8	43 (30; 46)	20 - 46	
Non-charitable health-related organizations (Ministry of Health, PAHO^††^, NGOs^‡‡^, etc.)	33.5 ±10.3	34.5 (25.5; 41.5)	10 - 50	
Work as a professor at the university or technical college level	35.9 ±9.4	35 (27; 44)	19 - 50	
Other	30.3 ±12.6	29 (20; 40)	10 - 50	

*IW-FLC= Intention to work in First-level care; ^†^SD = Standard deviation; ^‡^IQR = Interquartile range; ^§^Range = Minimum and maximum values; ^∥^Mann-Whitney U test result; ^¶^Kruskal-Wallis H-test result; **FLC = First-level care; ^††^PAHO = Pan American Health Organization; ^‡‡^NGOs = Non-governmental organizations

Regarding the identification of factors associated with the IW-FLC score, it was found that, among the independent variables, only the perception of the nurse’s work in FLC was significantly associated, increasing the median work intention score by 0.46 points (95% CI: 0.39, 0.53) as the PNW-FLC score increases (Table 5).

**Table 5:** Factors associated with the IW-FLC* score of nursing students at universities in Peru, 2023-2024 (n = 740)

Characteristics	Adjusted coefficient^†^ (95% CI^‡^)	P-value
PNW-FLC^§^ Scores	0.46 (0.39; 0.53)	<0.001
K-PHC^∥^ Scores	-0.02 (-0.22; 0.19)	0.866
Sociodemographic characteristics		
Gender		
Female	Reference	
Male	-2.51 (-4.63; -0.39)	0.020
Marital status		
Single	Reference	-
Married	1.46 (-2.87; 5.79)	0.507
In a common-law marriage	-0.62 (-4.42; 3.18)	0.749
Divorced	-7.18 (-15.13; 0.77)	0.077
Other	8.30 (-12.91; 29.5)	0.443
Have children		
No	Reference	
Yes	0.00 (-2.65; 2.64)	0.998
Economic dependence		
No	Reference	
Yes	0.54 (-2.00; 3.07)	0.677
Academic status		
Type of university		
Public	Reference	
Private	-2.16 (-3.92; -0.40)	0.016
Year of study		
Third	Reference	
Fourth	-0.44 (-3.07; 2.19)	0.742
Fifth	0.00 (-2.69; 2.69)	1.000
Alumnus (former student)	-3.60 (-7.28; 0.09)	0.056
Have you thought about changing careers?		
No	Reference	
Yes	-0.88 (-2.62; 0.86)	0.322
Previous education		
No	Reference	
Yes	-0.71 (-2.96; 1.55)	0.538
Frequency of internships at FLC^¶^		
None or very few	0.23 (-2.29; 2.74)	0.858
A few sessions	-0.59 (-2.77; 1.59)	0.594
Moderately frequent sessions	Reference	
Frequent sessions	-0.97 (-3.11; 1.18)	0.377
Very frequent sessions	0.40 (-2.47; 3.27)	0.785
Preparation for work in the FLC^¶^		
I am not prepared	-4.71 (-11.06; 1.63)	0.145
Somewhat prepared	-0.94 (-3.39; 1.50)	0.449
Generally prepared	Reference	
I am prepared	1.82 (0.02; 3.62)	0.048
I am very prepared	4.44 (0.67; 8.21)	0.021
Do you plan to pursue specialized studies?		
No	-7.48 (-13.81; -1.15)	0.021
I haven’t decided yet	Reference	
Yes	3.20 (0.65; 5.75)	0.014
Do you plan to pursue a master’s degree?		
No	-0.55 (-6.20; 5.10)	0.848
I haven’t decided yet	Reference	
Yes	0.40 (-1.81; 2.61)	0.723
Do you plan to pursue a doctoral degree?		
No	-2.29 (-6.61; 2.04)	0.299
I haven’t decided yet	Reference	
Yes	-0.76 (-2.55; 1.04)	0.409
Employment status		
Do you have a paid job?		
No	Reference	
Yes	0.05 (-2.57; 2.67)	0.970

*IW-FLC= Intention to work in first-level care; ^†^Adjusted Coef = Adjusted coefficient for quantile regression relative to the median; ^‡^95% CI = 95% confidence interval; ^§^PNW-FLC = Nurses’ perception of work in first-level care; ^∥^K-PHC= Knowledge of primary health care; ^¶^FLC = First-level care

Among the covariates studied, a negative trend was observed in the IW-FLC score, with male students having a median IW-FLC score that was 2.51 (95% CI: -4.63; -0.39) points lower than that of female students. On the other hand, students from private universities had a median IW-FLC score that was 2.16 (95% CI: -3.92; -0.40) points lower on the median IW-FLC score compared to students from public universities, while those who did not plan to pursue a specialization program had a median IW-FLC score that was 7.48 (95% CI: -13.81; -1.15) points lower compared to those who had not yet decided on such studies. The covariates that favored the IW-FLC score were readiness for work in FLC, with those who considered themselves prepared (1.82; 95% CI: 0.02; 3.62) and very prepared (4.44; 95% CI: 0.67; 8.21) having a higher median score compared to those who indicated they had regular training. Similarly, students planning to pursue specialized studies had IW-FLC scores that were 3.20 points (95% CI: 0.65; 5.75) higher compared to those who had not yet decided on such studies (Table 5). 

## Discussion

Nurses play a key role in FLC, as they are the only healthcare professionals available in many FLC territories and healthcare facilities, particularly in remote and rural areas^9^ ^,^ ^27^. However, the shortage of nurses is a critical problem and is likely to worsen due to potential departures from FLC and a trend away from this level as a career path.

The study revealed a notable IW-FLC among students, indicating a favorable attitude toward FLC based on positive normative beliefs regarding FLC and PC, such as identifying teachers involved in PHC policies as motivators and self-perceived readiness to work in FLC. These findings reflect progress in recognizing FLC as an area of professional development and PHC as a cornerstone of comprehensive care. Although recent literature in Latin America is scarce, the results are consistent with a study conducted in Peru that identified a greater willingness among nursing students to work in rural areas than among medical students^28^. In contrast, studies in Mexico (1.9%)^18^ and Peru (12.5%)^19^ reported very low IW-FLC. 

The perceptions of nurses’ work in FLC were mostly unfavorable, with negative assessments of prestige, working conditions, professional role within FLC, opportunities for professional development, leadership, and compensation. These results are consistent with a study conducted in Brazil^29^ that reported uncertainty between the ideal of PHC (connection, territory, comprehensiveness) and structural limitations. The complexity of the role of nursing in the FLC/PC was recognized through the combination of care, management and health promotion activities. Building bonds with users, knowledge of the territory, working with the community and an intercultural approach are core competencies. Besides, the administrative overload, the scarcity of resources and the difficulties in interdisciplinary work limit the full development of nursing practice at this level^29^.

Regarding knowledge of PHC, a moderate level prevailed, which is consistent with another study in Peru^30^ among health professionals. The assessment evaluated basic aspects of PHC (values, principles, elements) and the importance of PHC-based health systems^31^. These results call for a more robust integration of PHC into nursing education. On the other hand, the non-significant inverse relationship between knowledge and IW-FLC, along with the limited evidence from Latin America on this matter, highlights the need for further exploration in other countries. This assessment of the knowledge of soon-to-be-graduating students would allow for adjustments to nursing training curricula. In this context, the instrument designed and validated for Peru constitutes a valuable foundation for future adaptations and optimizations in other settings. 

The present study showed that positive perceptions of nursing work in FLC and self-perceived preparedness are associated with a greater intention of career in FLC. The fact of being male and attending a private university are negatively associated with this intention. These findings highlight the urgency of strengthening the training process in PHC. No prior studies were found among students. A study including with PC nurses in Brazil found that favorable perceptions of the work environment were associated with higher retention and job satisfaction^32^. These results suggest that improving students’ perceptions of the role of nurses in FLC may help attract them to this level, with this perception serving as a potential early predictor of career choice-an issue that must be addressed during training. These perceptions are dynamic and shaped by experiences, institutional environments, role models, and interactions with professors and tutors, and present significant challenges for education^33^ ^-^ ^34^ and underscore the need for reflection within educational institutions.

Given the shortage of nurses in Latin America, promoting a vocation for FLC/PHC is essential to reduce gaps and move toward equitable health systems and UHC. Universities and clinical settings are fundamental to training competent students with positive perceptions and attitudes toward FLC/PHC. In this regard, there is an urgent need for reforms and strategic actions in universities to consolidate nursing education focused on FLC/PHC^33^ ^-^ ^34^ through the training of upright and humanistic professionals with skills^31^ and tools that allow them to approach communities, respond to diversities, social determinants and community needs.

Countries are seeking to consolidate their health systems based on PHC, and universities must respond and contribute to this effort. Therefore, these reforms entail redesigning curricula with an explicit focus on PHC/FLC^35^, moving beyond the biomedical model^34^, reconceptualizing and transforming the meaning of nursing practices in the community^36^, promoting early and extended practice in communities and rural areas, strengthening the role of training, teaching, and tutor supervision^37^, and innovating strategies for interprofessional education, simulation^38^ ^-^ ^39^, and assessment of student perceptions. Evidence shows that educational programs with practical experience enhance competencies^34^, improve the assessment of the role of nursing in PC, and stimulate interest in the work. Nonetheless, there are still limitations in the curricula, with little community involvement and a lack of professional role models, which restrict understanding of the scope of nursing practice in PC^34^. 

The findings of this study provide useful evidence to guide labor policies that promote the recruitment and retention of nurses in FLC. Given their significant impact on “perception”, it is necessary to improve unfavorable working conditions and address perceived dissatisfaction within the FLC/PC and in community and rural settings^6^ ^,^ ^9^ ^,^ ^14^ ^,^ ^29^ ^,^ ^40^ ^-^ ^41^. Realities differ across and within countries, each with unique characteristics that require analysis. Effective governance of health systems is essential to strengthen FLC and PHC^42^, with increased investment in resources and FLC budgets, along with policies that can comprehensively adapt the structural and working conditions of nursing.

On the other hand, to improve “perceptions”, it is necessary to address administrative and care overload^27^, ensure adequate infrastructure, the availability of supplies, training, and postgraduate education, care protocols^43^, greater autonomy in care management^44^ ^-^ ^45^, and team management^46^. Moreover, social and organizational recognition of nurses’ roles should be promoted^13^ and psychosocial support provided to enhance their well-being. In the economic sphere, it is necessary to ensure stable contracts, fair compensation^13^ ^-^ ^14^, and establish differentiated incentives (salary scales, bonuses, benefits such as housing, transportation, meals, and others) to attract and retain nurses in FLC, especially in rural, Amazonian, and high-altitude areas to ensure the continuity and quality of comprehensive healthcare in these regions. 

Negative perceptions of the role of nursing in Primary Care Centers reflect a lack of professional recognition and devaluation, which align with the historically unfavorable social perception of nursing^6^ ^,^ ^9^ ^,^ ^14^ ^,^ ^40^ ^,^ ^47^. Such questions demand nursing organizations, universities, authorities, and nurses themselves to promote actions to reassess and achieve visibility^48^ of the impact of their work, strengthening professional identify and commitment^49^. The complexity and relevance in leading programs that impact the health of families and communities^12^, make FLC is a strategic space for positioning and empowering nursing^50^; however, it requires sustained improvements in working conditions and salaries for those at this level.

This study is the first to employ a quantitative approach among Peruvian students. We recommend conducting multicenter longitudinal studies that incorporate qualitative methods to better understand the facilitators and barriers to students’ career preferences in various contexts. Its main limitations are: a) The universities were selected for convenience, which could introduce selection bias and limit their representativeness; b) The study design was cross-sectional, and therefore it does not establish causal relationships; and c) It is likely that students’ limited experience and knowledge regarding the actual role of nurses in FLC may have influenced their perceptions or that they adjusted their responses due to social desirability. Therefore, the results should be interpreted with caution, taking these considerations into account.

## Conclusion

Positive perceptions of nursing work in FLC positively influence nursing students’ intention to work at this level. On the other hand, knowledge of PHC does not appear to influence the intention to work. However, further studies are recommended, given that the bivariate analysis suggested that greater PHC knowledge was associated with lower intention to work in FLC. 

The self-perception of being prepared to work in FLC has a positive influence on interest in pursuing careers at this level, unlike among students from private universities and male students. The prevalence of students with a moderate level of knowledge about PHC calls for a more robust integration of PHC into nursing education.

The present findings highlight the importance of addressing perceptions of the image and role of nursing in FLC during university education, fostering sound judgment and to acquire an appropriate understanding of nursing that encourages a career in FLC. Additionally, to reverse the unfavorable perceptions identified and addressing the high proportion of students with only a basic level of knowledge about PHC.

On the other hand, it is necessary to promote strategies for the reassessment, visibility and empowerment of nursing in the PNA, as well as for the defense of the improvement of working conditions in the PNA and the strengthening of the PNA, which allow for the reversal of the adverse reality it faces, making work at this level more attractive.

## Data Availability

Datasets related to this article will be available upon request to the corresponding author.
