# Supplementary material for: Perceptions and knowledge in primary health care and career intention at the first level of care: a cross-sectional study
Source: Rev Lat Am Enfermagem. 2026 Oct 23;34:e4973. doi: 10.1590/1518-8345.8021.4973 (PMC13706264; doi:10.1590/1518-8345.8021.4973)
Supplement: Translation

# Translation: Percepções e conhecimento em atenção primária à saúde e intenção de carreira no primeiro nível de atenção: um estudo transversal^*^

**DOI:** 10.1590/1518-8345.8021.4974

**Authors:** Maricela Curisinche-Rojas, Tula Margarita Espinoza-Moreno, Reyna Ysmelia Peralta-Gómez, Lucy Zenelia Barrantes-Cruz, Gilmer Solis-Sánchez, Irma Yupanqui-Martinez, Lida Inés Carhuas-Peña, Cindy Vargas-Cabrera, Juvita Dina Soto-Hilario, Tomás Caycho-Rodriguez

**Affiliations:** 1 Universidad Científica del Sur, Facultad de Enfermería, Lima, Peru.; 2 Universidad Nacional Mayor de San Marcos, Lima, Peru.; 3 Universidad Nacional de San Agustín, Departamento Académico de Enfermería, Arequipa, Peru.; 4 Universidad Privada Antenor Orrego, Trujillo, La Libertad, Peru.; 5 Universidad Científica del Sur, Lima, Peru.; 6 Universidad Nacional Daniel Alcides Carrión, Facultad de Ciencias de la Salud-Escuela de Formación Profesional de Enfermería, Cerro de Pasco, Pasco, Peru.; 7 Universidad Nacional de Huancavelica, Facultad de Enfermería, Huancavelica, Peru.; 8 Universidad Señor de Sipán, Escuela de Enfermería, Chiclayo, Lambayeque, Peru.; 9 Universidad Nacional Hermilio Valdizán, Huánuco, Peru.

**Keywords:** Enfermagem, Atenção Primária à Saúde, Percepção, Conhecimento, Intenção, Trabalho.

## Abstract

**Objetivo:**

determinar a associação entre a percepção do trabalho de enfermagem e o conhecimento sobre atenção primária de saúde com a intenção de carreira no primeiro nível de atenção.

**Método:**

estudo transversal analítico, realizado com 740 estudantes universitários de enfermagem. A intenção de carreira no primeiro nível de atenção, a percepção do trabalho de enfermagem e os conhecimentos sobre cuidados de saúde primários foram avaliados utilizando instrumentos ad hoc validados. Realizaram-se uma análise bivariada e análise de regressão quantílica mediana.

**Resultados:**

predominaram elevada intenção de carreira (66,8%), percepção pouco favorável do trabalho de enfermagem (53,0%) e nível moderado de conhecimentos (67,0%). A intenção de carreira foi positivamente associada à percepção do trabalho (coeficiente ajustado: 0,46, IC 95%: 0,39; 0,53), com a autopercepção da preparação para o trabalho e com o planejamento de uma especialização. O sexo masculino e a intenção de estudar em universidades privadas foram associados negativamente. Não foi encontrada associação significativa com o conhecimento.

**Conclusão:**

percepções favoráveis do trabalho de enfermagem no primeiro nível de atenção aumentam a intenção de carreira nesse nível, enquanto o conhecimento sobre atenção primária à saúde não teria influência. Abordar a percepção do trabalho durante a educação universitária é essencial para promover o interesse profissional dos futuros enfermeiros.

## Destaques

**(1)** Futuros enfermeiros na atenção primária ajudarão a reduzir lacunas na enfermagem.

**(2)** A percepção favorável da enfermagem aumenta a intenção de carreira em atenção primária.

**(3)** O nível médio de conhecimento sobre atenção primária à saúde foi predominante.

**(4)** Em universidades privadas e entre os indivíduos do sexo masculino, a intenção de carreira na atenção primária é menor.

**(5)** A academia deve construir uma visão positiva da enfermagem no primeiro nível de atenção.

## Introdução

 “Não deixar ninguém para trás” e alcançar a cobertura universal de saúde (CUS) no mundo estão cada vez mais distantes. Enfrentamos um preocupante panorama global de estagnação na CUS, com mais de 50% da população mundial sem cobertura total de serviços de saúde essenciais e muitas famílias enfrentando despesas catastróficas^1^. Nos países da América Latina, ainda existem altas taxas de necessidades de saúde não atendidas e a presença de várias barreiras ao acesso que limitam o desempenho da atenção primária à saúde (APS)^2^ ^-^ ^3^. Atualmente, mostra-se premente consolidar os sistemas de saúde com foco na APS como eixo central de sua organização e gestão. A APS é a estratégia mais inclusiva, pró-equidade, econômica e eficiente para superar barreiras de acesso, aumentar a proteção financeira das famílias e avançar em direção aos Objetivos de Desenvolvimento Sustentável (ODS)^1^ ^-^ ^2^. No Peru, são evidentes os esforços e avanços para posicionar a APS como a base do sistema de saúde; no entanto, passar de um modelo conceitual para uma implementação autêntica ainda constitui um processo lento^4^.

O primeiro nível de atenção (PNA) é essencial para tornar eficazes os atributos da APS (primeiro contato, longitudinalidade, abrangência e coordenação). Constitui a porta e a base da pirâmide da saúde, com ampla presença em comunidades rurais e urbanas marginais, e é integrado e articulado com outros níveis de cuidado para garantir a continuidade do cuidado. Este nível oferece atenção primária (AP), que inclui cuidados de saúde abrangentes ao longo da vida para o indivíduo, família e comunidade. Além disso, há promoção da saúde e prevenção de riscos e danos em corresponsabilidade com a comunidade organizada e o governo local para enfrentar os determinantes sociais da saúde^5^. No entanto, o PNA enfrenta sérios problemas que limitam seu desempenho^2^ ^,^ ^6^, sendo a escassez de enfermeiros um fator crítico^7^ ^-^ ^8^.

As enfermeiras são a principal força de trabalho em saúde e desempenham um papel fundamental no PNA e na APS^8^, principalmente em áreas rurais e menos desenvolvidas^9^. Dado seu amplo perfil profissional e visão salutogênica, são especialistas no cuidado integral da pessoa, da família e da comunidade^10^. A prática da enfermagem como essência da APS^7^, sua liderança e autonomia na saúde, e sua responsabilidade social, ética e humanista em relação à APS, são amplamente relatadas globalmente^7^ ^,^ ^11^ e na América Latina^9^ ^,^ ^12^. No entanto, na América Latina, enfermeiros do PNA enfrentam condições de trabalho desfavoráveis que limitam a prática da AP e influenciam negativamente a satisfação no trabalho e a inclinação para permanecer no PNA^6^ ^,^ ^9^ ^,^ ^13^ ^-^ ^14^ **,** semelhante ao que foi observado em outras regiões^15^. Escassez e distribuição desigual de enfermeiros, sobrecarga de trabalho e administrativa, longos turnos, recursos escassos, infraestrutura inadequada, estresse no trabalho, reconhecimento insuficiente, insegurança no emprego, entre outros^6^ ^,^ ^9^ ^,^ ^13^ ^-^ ^14^ **,** se destacam, e essa situação não é estranha à realidade peruana^16^. A ausência ou rotação de enfermeiros no PNA afeta o acesso à AP^17^ **,** e a atração e retenção desses profissionais no PNA é fundamental; as evidências indicam que está ligada a condições estruturais ótimas na prática, percepções favoráveis e bem-estar no trabalho^15^.

Os estudantes representam a reserva estratégica futura da enfermagem, que contribuirá para preencher lacunas na AP. Portanto, é prioridade estar atento às expectativas e intenções de carreira desses estudantes, além de explorar fatores que facilitam ou limitam. Na América Latina, há poucos precedentes nesse sentido. Dois estudos no México e no Peru notaram baixas expectativas de trabalho no PNA e preferências pela área hospitalar^18^ ^-^ ^19^; no entanto, eles não aprofundaram a análise dos fatores ou explicações para essa tendência, o que ressalta a necessidade de estudos nesse sentido.

Evidências em outros territórios de alcance global alertam sobre baixas preferências de trabalho em ambientes da AP por estudantes de enfermagem^20^ ^-^ ^23^ e que percepções inadequadas da APS e do papel da enfermagem, em relação a prestígio, salário e condições de trabalho, atuam como barreiras para a escolha do PNA/AP como área profissional. Em contraste, origem rural, autonomia e exposição curricular adequada são reconhecidas como fatores facilitadores^20^ ^-^ ^23^. No Peru, desconhece-se o que está ocorrendo nesse sentido.

Nesse contexto, esse estudo fundamenta-se no princípio de que estudantes de enfermagem peruanos próximos ou recém-formados com percepções favoráveis do papel da enfermagem no PNA, bem como aqueles que possuem conhecimentos adequados sobre a APS adquiridos durante sua formação profissional, teriam maior intenção de carreira na APS. Nesse sentido, a questão que orientou a pesquisa foi: a percepção do trabalho da enfermeira no PNA e o conhecimento sobre a APS estão associados à intenção de carreira no PNA de estudantes de enfermagem de universidades peruanas?

O objetivo deste estudo foi determinar a associação entre a percepção do trabalho do enfermeiro no PNA e o conhecimento sobre a APS, com a intenção de carreira no PNA dos estudantes de enfermagem no Peru. As evidências geradas servirão como suporte na tomada de decisões para aprimorar os planos curriculares e os processos de treinamento, planejamento e alinhamento da oferta de treinamento com a real demanda do sistema de saúde, programas de motivação e incentivo para atrair e reter jovens talentos no PNA e políticas para fortalecer o PNA.

## Método

### Delineamento do estudo 

Realizou-se um estudo observacional, transversal e analítico, seguindo as diretrizes do *Strengthening the Reporting of Observational Studies in Epidemiology* (STROBE). 

### Local e período de coleta de dados

O estudo foi realizado em 11 universidades públicas e privadas do Peru que oferecem carreiras de enfermagem, localizadas em quatro macrozonas do país: norte, centro, sul e Lima Metropolitana. Essa distribuição possibilitou a presença de representantes de territórios com diferentes realidades sociais, de treinamento e de saúde.

A viabilidade operacional do estudo (autorização institucional e ter um copesquisador da universidade) e o tamanho da população estudantil foram critérios para a seleção das universidades.

No Norte, foram incluídas duas universidades localizadas em cidades costeiras com alta concentração urbana (Trujillo e Chiclayo), com serviços de saúde presentes em contextos urbanos e rurais. Na região central, foram incluídas três universidades de áreas andinas (Huancavelica, Huánuco e Cerro de Pasco). Essas regiões são caracterizadas por populações predominantemente rurais e dispersas, maiores índices de pobreza e alta concentração de centros de saúde do PNA. No Sul, foi selecionada uma universidade em Arequipa, a segunda maior cidade do país e polo regional, com serviços altamente especializados e de primeiro nível. Por fim, em Lima Metropolitana, capital do país e principal centro acadêmico e de emprego na área da saúde, caracterizada por sua grande população estudantil e diversidade institucional, foram escolhidas cinco universidades.

Selecionaram-se universidades públicas e privadas, considerando que ambas representam a diversidade existente no ensino superior na enfermagem, com diferenças nas condições de formação acadêmica e nas perspectivas de acesso ao mercado de trabalho de acordo com o tipo de gestão institucional.

A coleta de dados foi realizada entre novembro de 2023 e fevereiro de 2024.

### População, critérios de seleção e amostra

A população era composta por estudantes de enfermagem matriculados entre o terceiro e o quinto ano (último ano) e formados no ano de 2022 das universidades selecionadas. Foi dada prioridade a estudantes de ciclos avançados devido à maior exposição curricular teórico-prática à APS/PNA, e aos recém-formados, para integrar a perspectiva da transição para o mercado de trabalho.

O tamanho da amostra não foi estimado; participaram todos os alunos que atenderam aos critérios de elegibilidade, que deveriam ter 18 anos ou mais e que deram consentimento informado. Excluíram-se estudantes com desistência temporária ou ausência nas datas da pesquisa.

### Variáveis

As principais variáveis foram a intenção de carreira no primeiro nível de atenção (IT-PNA), a percepção do trabalho de enfermagem no primeiro nível de atenção (PTE-PNA) e o conhecimento sobre atenção primária à saúde (C-APS), que foram medidas numericamente por meio de escores obtidos em escalas do tipo Likert. O PTE-PNA e o IT-PNA foram medidos de forma geral e por cada uma de suas dimensões; além disso, a distribuição das pontuações obtidas em três níveis foi categorizada por intervalos de classe.

Coletaram-se informações sobre outras covariáveis de acordo com as características dos entrevistados: i) sociodemográfica, ii) acadêmica e iii) trabalhista. No nível sociodemográfico, registraram-se idade, sexo, estado civil, se tinham filhos (sim/não) e dependência econômica; enquanto as características acadêmicas avaliadas foram o tipo de universidade, a região do país onde a universidade estava localizada, o ano de estudo, caso se pensasse em mudar de carreira, os estudos anteriores (em nível técnico ou universitário), a frequência dos estágios no PNA, a preparação para o trabalho no PNA e planos de especialização, mestrado e/ou doutorado. No nível do trabalho, registrou-se se tinham trabalho remunerado, setor e local de trabalho e onde planejavam trabalhar. 

### Instrumentos de coleta de dados

PTE-PNA, IT-PNA e C-APS foram medidos por meio de escalas ad hoc construídas especificamente para este estudo. As escalas para medir o PTE-PNA e IT-PNA consistiam em declarações ou itens com cinco alternativas de resposta do tipo Likert: Não concordo, Concordo parcialmente, Concordo moderadamente, Concordo, Concordo totalmente; que foram pontuadas de 1 a 5, respectivamente. Por outro lado, a escala para medir o C-APS era composta por afirmações de escolha verdadeira-falsa, atribuindo uma pontuação de 0 quando a resposta estava errada e 1 quando estava correta.

A escala proposta para o PTE-PNA consistia em 13 declarações distribuídas em três dimensões: 1 - Imagem da enfermeira que atua no PNA, 2 - Trabalho no PNA, e 3 - Consequências econômicas do trabalho no PNA. Por outro lado, a escala para a medição da IT-PNA consistia em 10 afirmações distribuídas em três dimensões: 1 - Atitude para trabalhar no PNA, 2 - Crenças normativas subjetivas e 3 - Crenças ou autopercepção da capacidade de controlar o comportamento. A escala C-APS possuía 31 afirmações, sem distribuição nas dimensões. Além disso, utilizou-se um questionário específico para características sociodemográficas, acadêmicas e laborais.

### Validação de instrumentos de coleta de dados

A elaboração da escala IT-PNA foi fundamentada na Teoria do Comportamento Planejado^24^ ^-^ ^25^ ^)^ e as declarações para cada dimensão foram formuladas com base em uma revisão da literatura. Em relação à escala C-APS, foram consideradas declarações sobre os valores, princípios e elementos da APS e a importância do sistema de saúde baseado na APS, identificadas por meio de revisão da literatura científica e documentos técnicos. Por outro lado, a escala PTE-PNA resultou da reestruturação e adaptação ao contexto de enfermagem de uma escala utilizada em estudantes de medicina na América Latina^26^.

As três escalas foram validadas por 11 juízes especialistas compostos por enfermeiros especialistas, com mestrado e/ou doutorado em saúde pública, comunitária e/ou familiar. Além disso, possuíam tempo de experiência acima de 10 anos no PNA e/ou na gestão de programas de saúde em nível regional ou nacional; eram procedentes de Lima e das regiões envolvidas no estudo.

Por meio de um formulário online compartilhado, os jurados desenvolveram as matrizes de validação dos itens de cada escala, considerando critérios de relevância, clareza e relevância. Os resultados foram analisados usando o coeficiente V de Aiken, obtendo-se graus aceitáveis de concordância em todas as afirmações (itens), para o PTE-PNA entre 0,75 e 1,00, para o IT-PNA entre 0,73 e 0,97 e para o C-APS entre 0,73 e 1,00.

Determinou-se a confiabilidade dos instrumentos pela consistência interna, usando o coeficiente Ômega de McDonald’s; constatou-se que, para cada dimensão do PTE-PNA, ela era 0,908 (D1), 0,827 (D2) e 0,834 (D3); enquanto para as dimensões do IT-PNA eram 0,855 (D1), 0,914 (D2) e 0,880 (D3); por sua vez, para o C-APS esse coeficiente era 0,794.

Devido à distribuição das afirmações da PTE-PNA e IT-PNA em dimensões, a validade do construto foi realizada por análise fatorial confirmatória, encontrando índices de ajuste aceitáveis para ambas as escalas (p<0,001); especificamente para o PTE-PNA, foram encontrados um *Comparative Fit Index* (CFI) de 0,963, um *Root Mean Square Error of Approximation* (RMSEA) de 0,080 (intervalo de confiança de 95% [IC 95%]: 0,070; 0,102) e um *Standardized Root Mean Square Residual* (SRMR) de 0,03. Quanto à escala IT-PNA, o CFI foi 0,950, o RMSEA foi 0,117 (IC 95%: 0,103; 0,131) e o SRMR foi 0,03.

### Coleta de dados

Antes de iniciar a coleta de dados, foram realizadas reuniões presenciais e virtuais para coordenar e organizar as atividades do estudo. Da mesma forma, por meio de sessões virtuais, os responsáveis pela coleta foram treinados nos procedimentos para obter consentimento informado e aplicar os instrumentos.

A coleta de dados foi realizada por copesquisadores docentes de cada universidade, que gerenciaram as autorizações, coordenaram as datas e reuniram os participantes em espaços institucionais. Após verificação dos critérios de elegibilidade, os instrumentos foram aplicados anonimamente, sem registro de nomes ou sobrenomes.

No caso de graduados e estudantes de 2022 que não participaram das reuniões presenciais, utilizou-se uma versão digital dos instrumentos no Google *Forms*, compartilhada por e-mail ou WhatsApp, sendo feito, então, o acompanhamento, com envio de lembretes para maximizar a taxa de resposta.

### Processamento e análise de dados

Os dados coletados foram organizados em uma planilha MS Excel e, em seguida, analisados por meio de estatísticas descritivas e inferenciais, utilizando-se os programas STATA v. 17.0 (Stata Corporation, College Station, Texas, EUA), os quais possibilitaram estimar as medidas de frequência absoluta (n) e relativas (%) das variáveis qualitativas coletadas. Identificou-se a distribuição absoluta e relativa da frequência das respostas a cada pergunta dos três (03) questionários aplicados.

As pontuações obtidas a partir da soma de cada dimensão e da escala total para cada um dos questionários foram avaliadas por medidas de tendência central (média e mediana) e dispersão (intervalo, desvio-padrão, intervalo interquartílico). A partir das pontuações de cada escala, três intervalos de classe foram gerados pela regra de Sturges, categorizados como Desfavoráveis, Desfavoráveis e Favoráveis para o PTE-PNA; enquanto para IT-PNA e C-APS, as categorizações foram: Baixo, Moderado e Alto. Esses intervalos eram realizados de forma geral e para cada dimensão da escala.

Avaliou-se a diferença na pontuação do questionário de intenção de carreira para cada uma de suas categorias e na escala total, de acordo com as categorias dos intervalos de classe, as dimensões e a escala total dos questionários de conhecimento em atenção primária à saúde e a percepção do trabalho do enfermeiro no PNA, bem como de acordo com as categorias do estudo, covariáveis. Essa comparação foi feita usando o teste t de Student para amostras independentes ou Mann-Whitney U e a Análise de Variância de um fator (ANOVA) ou Kruskal-Wallis H. A escolha dos testes baseou-se na identificação da distribuição normal ou não normal (teste de Shapiro-Wilk) e no número de grupos a serem comparados (2, 3 ou mais).

A relação entre a pontuação do questionário de intenção de carreira com as pontuações dos questionários de conhecimento em atenção primária à saúde e a percepção do trabalho do enfermeiro no PNA foi identificada usando os testes de coeficiente de correlação de Pearson ou Spearman, de acordo com a distribuição normal ou não normal (teste de Shapiro-Wilk). Para avaliar os fatores associados à pontuação do questionário de intenção de carreira, foi realizada uma regressão quantílica mediana ajustada, considerando como variáveis de ajuste as variáveis independentes (pontuação dos questionários de conhecimento em atenção primária e percepção do trabalho do enfermeiro no PNA) e as covariáveis que apresentaram valor p menor que 0,20 no modelo bruto; variâncias robustas foram consideradas para evitar afetar o modelo devido a erros causados pela heterocedasticidade no modelo. Todos os resultados foram trabalhados com um nível de significância de 0,05.

### Aspectos éticos

Antes de iniciar o estudo, o projeto de pesquisa foi aprovado pelo Comitê de Ética em Pesquisa Institucional da Universidad Científica del Sur (Certificado n.º 055-CIEI- Científica-2023) com autorização obtida pelas universidades participantes. Antes da aplicação dos instrumentos, foram fornecidas informações detalhadas sobre o estudo, bem como o consentimento informado assinado dos estudantes que concordaram em participar do estudo. Em relação à coleta virtual de dados, o instrumento digital (Google *Forms*) teve uma primeira seção com uma explicação detalhada do estudo, sobre aspectos relacionados à participação do estudante no estudo e um consentimento eletrônico no qual eles escolheriam entre participar ou não do estudo. Os estudantes que concordaram em participar desenvolveram os instrumentos voluntariamente.

Os dados pessoais eram protegidos por meio de mecanismos de anonimização, acessíveis apenas aos pesquisadores.

## Resultados

Dos 740 estudantes de enfermagem que participaram do estudo, constatou-se que a média de idade era de 24,4 ±4,9 anos, 85% eram do sexo feminino, 89,2% eram solteiras, 84,2% sem filhos, 74,3% eram economicamente dependentes, 76,4% não possuíam trabalho remunerado, 59,5% estudavam em universidades públicas, 44,2% estudavam em universidades em Lima, 44,5% estavam no quarto ano de estudo, 71,9% não planejavam mudar de carreira e 80,0% não possuíam formação superior prévia. Em relação à sua formação, 32,0% realizaram estágios com frequência moderada, 44,3% relataram estar preparados para atuar na PNA, 64,3% indicaram intenção de carreira em um “Hospital, Clínica, Instituto Especializado”, 87,4% planejavam estudos de especialização, 77,4% planejavam estudos de mestrado e 53,6% planejavam cursar o doutorado (Tabela 1).

**Tabela 1:** Características dos estudantes de enfermagem pesquisados no Peru, 2023-2024 (n = 740)

Características	n | Média	% | DP*
Sociodemográficas		
Idade (Média ±DP*)	24,4	4,9
Sexo		
Feminino	629	85,0
Masculino	111	15,0
Estado civil		
Solteiro	660	89,2
Casado	31	4,2
Em união estável	41	5,5
Divorciado	7	0,9
Outros	1	0,1
Possuir filhos		
Não	623	84,2
Sim	117	15,8
Dependência econômica		
Não	190	25,7
Sim	550	74,3
Aspectos acadêmicos		
Tipo de universidade		
Pública	440	59,5
Privada	300	40,5
Área do país no qual a universidade está localizada		
Centro	178	24,1
Norte	106	14,3
Sul	129	17,4
Lima	327	44,2
Ano de estudo		
Terceiro	80	10,8
Quarto	329	44,5
Quinto	264	35,7
Graduado (ex aluno)	67	9,1
Pensou em mudar de carreira		
Não	532	71,9
Sim	208	28,1
Estudos anteriores		
Não	592	80,0
Sim	148	20,0
No nível técnico	119	80,4
No nível universitário	29	19,6
Frequência de práticas no PNA^†^		
Não houve ou foram pouquíssimas práticas	103	13,9
Poucas práticas	149	20,1
Práticas moderadamente frequentes	237	32,0
Práticas frequentes	174	23,5
Práticas muito frequentes	77	10,4
Preparação para o trabalho no PNA^†^		
Não estou preparado	12	1,6
Um pouco preparado	102	13,8
Regularmente preparado	256	34,6
Estou preparado	328	44,3
Estou muto preparado	42	5,7
Planeja realizar estudos de especialização		
Não	13	1,8
Ainda não decidi	80	10,8
Sim	647	87,4
Planeja cursar o mestrado		
Não	21	2,8
Ainda não decidi	146	19,7
Sim	573	77,4
Planeja cursar o doutorado		
Não	35	4,7
Ainda não decidi	308	41,6
Sim	397	53,6
Situação profissional		
Possui trabalho remunerado		
Não	565	76,4
Sim	175	23,6
Setor onde trabalha (n=175)		
Trabalha em outros setores	67	38,3
Trabalha na área de saúde	108	61,7
Local de trabalho remunerado (n=175)		
Centro ou posto de saúde	27	15,4
Hospital, clínica ou instituto especializado	81	46,3
Outros setores não relacionados à saúde	67	38,3
Onde pretende atuar		
Hospital, Clínica, Instituto especializado	478	64,6
Centro ou posto de saúde do PNA^†^	153	20,7
Instituições dedicadas à pesquisa pública ou privada	31	4,2
Organizações não assistenciais relacionadas à saúde (Ministério da Saúde, OPS^‡^, ONGs^§^, etc)	40	5,4
Trabalhar como professor em nível universitário ou técnico	23	3,1
Outros	15	2,0

*DP = Desvio-Padrão; ^†^PNA = Primeiro nível de atenção; ^‡^OPS = Organização Panamericana de Saúde; ^§^ONGs = Organizações não Governamentais

De acordo com as respostas obtidas nos questionários, a pontuação mediana para as avaliações IT-PNA e PTE-PNA foi de 39 (RIC: 30 - 39); enquanto a pontuação mediana para C-APS foi de 18 (RIC: 16 - 18). As pontuações foram classificadas de acordo com o nível, constatando-se que 66,8% obtiveram elevada avaliação para IT-PNA, 53,0% obtiveram percepção desfavorável do trabalho da enfermeira na PNA e 67,0% dos estudantes alcançaram nível moderado de conhecimento sobre a APS (Tabela 2).

A relação entre a pontuação IT-PNA e a PTE-PNA mostrou uma correlação estatisticamente significativa (p<0,001) com um valor de Rho de 0,531; a relação entre a pontuação IT-PNA e o C-APS mostrou uma correlação estatisticamente significativa inversamente com um valor de Rho de -0,166 (Tabela 3).

As pontuações de IT-PNA variaram de acordo com as categorias de algumas variáveis (p<0,05), com pontuações mais elevadas encontradas em estudantes do sexo feminino, com dependência econômica, estudando em universidade pública, no terceiro ano, sem pensar em mudar de carreira, indicando que os estágios no PNA eram muito frequentes, consideram que estão bastante preparados para trabalhar no PNA. Em relação à intenção de estudos futuros, tanto aqueles que pretendiam cursar especialização ou mestrado apresentaram pontuações mais elevadas no IT-PNA, quanto para a intenção de estudos de doutorado, as maiores pontuações foram daqueles que ainda não haviam decidido realizar tais estudos (Tabela 4).

**Tabela 2:** Pontuações e níveis obtidos na avaliação de IT-PNA*, PTE-PNA^†^ e C-APS^‡^ em estudantes de enfermagem no Peru, 2023-2024 (n = 740)

Variáveis	Pontuações			Nível n (%)		
	Média ±DP^§^	Mediana (RIC^∥^)	Intervalo^¶^	Baixo / Desfavorável**	Moderado / Pouco favorável**	Alto / Favorável**
IT-PNA*						
Dimensão: Atitude em relação à carreira no PNA^††^	14,3 ±3,7	16 (12 - 16)	4 - 20	99 (13,4)	206 (27,8)	435 (58,8)
Dimensão: Crenças normativas subjetivas (pressão social)	11,5 ±3,2	12 (9 - 12)	3 - 15	109 (14,7)	188 (25,4)	443 (59,9)
Dimensão: Crenças ou autopercepção da capacidade de controle do comportamento	11,2 ±3,1	12 (9 - 12)	3 - 15	105 (14,2)	239 (32,3)	396 (53,5)
Avaliação geral da IT-PNA*	36,9 ±9,3	39 (30 - 39)	10 - 50	28 (3,8)	218 (29,5)	494 (66,8)
PTE-PNA^†^						
Dimensão: Imagem da enfermeira (o) atuando no PNA^††^	14,4 ±5,1	15 (10 - 15)	5 - 25	250 (33,8)	276 (37,3)	214 (28,9)
Dimensão: O trabalho no PNA^††^	19,0 ±5,3	20 (16 - 20)	6 - 30	113 (15,3)	327 (44,2)	300 (40,5)
Dimensão: Consequências econômicas referentes ao trabalho no PNA^††^	5,5 ±2,0	6 (4 - 6)	2 - 10	551 (74,5)	144 (19,5)	45 (6,1)
Avaliação geral da PTE-PNA^†^	38,9 ±11,3	39 (30 - 39)	13 - 65	76 (10,3)	392 (53,0)	272 (36,7)
C-APS^‡^						
Avaliação geral do C-APS^‡^	19,0 ±3,8	18 (16 - 18)	10 - 30	0 (0,0)	496 (67,0)	244 (33,0)

*IT-PNA = Intenção de carreira no primeiro nível de atenção; ^†^PTE-PNA = Percepção do trabalho da enfermeira no primeiro nível de atenção; ^‡^C-APS = Conhecimentos sobre atenção primária de saúde; ^§^DP = Desvio-Padrão; ^∥^RIC = Intervalo Interquartílico; ^¶^Intervalo = Valor mínimo e máximo; **Valor aplicável para PTE-PNA; ^††^PNA = Primeiro nível de atenção

**Tabela 3:** Avaliação da correlação da pontuação IT-PNA* com as pontuações de percepção PTE-PNA^†^ e C-APS^‡^ em estudantes de enfermagem no Peru, 2023-2024 (n = 740)

	Rho^§^	Valor P
PTE-PNA^†^		
Dimensão: Imagem da enfermeira (o) trabalhando no PNA^∥^	0,508	<0,001
Dimensão: O trabalho no PNA^∥^	0,485	<0,001
Dimensão: Consequências econômicas referentes ao trabalho no PNA^∥^	0,408	<0,001
Avaliação geral da PTE-PNA^†^	0,531	<0,001
C-APS^‡^		
Avaliação do C-APS^‡^	-0,166	<0,001

*IT-PNA = Intenção de carreira no primeiro nível de atenção; ^†^PTE-PNA = Percepção do trabalho da enfermeira no primeiro nível de atenção; ^‡^C-APS = Conhecimentos sobre atenção primária de saúde; ^§^Rho = Coeficiente Rho de Spearman; ^∥^PNA = Primeiro nível de atenção

**Tabela 4:** Pontuações IT-PNA* de acordo com características dos estudantes de enfermagem no Peru, 2023-2024 (n = 740)

Características	Pontuação de IT-PNA*			Valor P
	Média ±DP^†^	Mediana (RIC^‡^)	Intervalo^§^	
Sociodemográficas				
Sexo				
Feminino	37,3 ±9,1	40 (31; 46)	10 - 50	0,028^∥^
Masculino	34,7 ±10,4	38 (27; 43)	10 - 50	
Estado civil				
Solteiro	37,2 ±9,0	39 (31; 45)	10 - 50	0,067^¶^
Casado	37,8 ±11,0	40 (27; 47)	10 - 50	
Em união estável	33,0 ±12,3	33 (21; 45)	10 - 50	
Divorciado	29,3 ±10,5	27 (26; 30)	15 - 50	
Outros	3,00 ±0,0	30 (30; 30)	30 - 30	
Ter filhos				
Não	37,2 ±9,0	40 (31; 45)	10 - 50	0,148^∥^
Sim	35,3 ±10,7	39 (26; 45)	10 - 50	
Dependência econômica				
Não	35,0 ±10,0	37,5 (27; 43)	10 - 50	0,002^∥^
Sim	37,6 ±9,0	40 (32; 46)	10 - 50	
Aspectos acadêmicos				
Tipo de universidade		-		
Pública	37,8 ±8,9	40 (32; 46)	10 - 50	0,003^∥^
Privada	35,7 ±9,8	39 (28; 43)	10 - 50	
Ano de estudo				
Terceiro	40,0 ±9,0	45,5 (36; 46)	14 - 50	0,001^¶^
Quarto	36,5 ±9,5	39 (30; 44)	10 - 50	
Quinto	37,0 ±8,9	39 (31,5; 44)	10 - 50	
Graduado (ex-aluno)	35,3 ±9,7	37 (28; 43)	10 - 50	
Pensou em mudar de carreira				
Não	37,7 ±9,3	40 (31; 46)	10 - 50	<0,001^∥^
Sim	35,1 ±9,1	37 (28; 41)	10 - 50	
Estudos anteriores				
Não	37,3 ±9,1	40 (31; 46)	10 - 50	0,096^∥^
Sim	35,6 ±10,1	38 (27,5; 44)	10 - 50	
Estudos anteriores concluídos				
Não	37,3 ±9,1	40 (31; 46)	10 - 50	0,249^¶^
No nível técnico	35,5 ±10,2	38 (27; 44)	10 - 50	
No nível universitário	35,7 ±9,7	39 (30; 41)	10 - 50	
Frequência de práticas no PNA**				
Não houve ou foram muito poucas	36,6 ±9,4	39 (30; 45)	10 - 50	<0,001^¶^
Poucas práticas	35,0 ±8,9	38 (28; 40)	13 - 50	
Práticas moderadamente frequentes	38,0 ±9,5	40 (32; 46)	10 - 50	
Práticas frequentes	36,4 ±9,1	39 (30; 42)	10 - 50	
Práticas muito frequentes	39,0 ±9,4	41 (34; 46)	14 - 50	
Preparação para o trabalho no PNA**				
Não estou preparada	31,1 ±12,0	33 (24,5; 40)	10 - 49	<0,001^¶^
Algo preparada	33,4 ±9,5	35 (25; 40)	13 - 50	
Regularmente preparada	35,7 ±8,9	38 (30; 42)	10 - 50	
Estou preparada	38,8 ±8,9	40 (34; 46)	10 - 50	
Estou bastante preparada	40,5 ±9,9	44 (33; 49)	20 - 50	
Planeja realizar estudos de especialização				
Não	26,5 ±12,5	24 (20; 36)	10 - 48	<0,001^¶^
Ainda não decidi	33,8 ±9,2	35 (26; 40)	10 - 50	
Sim	37,5 ±9,1	40 (31; 46)	10 - 50	
Planeja cursar o mestrado				
Não	30,8 ±12,3	31 (22; 41)	10 - 48	0,009^¶^
Ainda não decidi	36,1 ±8,8	38,5 (29; 42)	13 - 50	
Sim	37,4 ±9,3	40 (31; 46)	10 - 50	
Planeja cursar o doutorado				
Não	30,2 ±10,8	31 (23; 39)	10 - 47	<0,001^¶^
Ainda não decidi	37,9 ±9,3	40 (31; 46)	13 - 50	
Sim	36,8 ±9,0	39 (30; 43)	10 - 50	
Situação profissional				
Possui trabalho remunerado				
Não	37,4 ±9,0	40 (31; 46)	10 - 50	0,151^∥^
Sim	35,3 ±10,2	37 (28; 43)	10 - 50	
Setor onde trabalha (n=175)				
Trabalha em outros setores	36,7 ±9,5	39 (30; 45)	10 - 50	0,151^∥^
Trabalha na área de saúde	34,4 ±10,5	35 (26; 42)	10 - 50	
Onde planeja trabalhar				
Hospital, Clínica, Instituto especializado	36,3 ±9,3	39 (30; 44)	10 - 50	<0,001^¶^
Centro ou posto de saúde do PNA**	40,3 ±8,0	41 (38; 46)	13 - 50	
Instituições destinadas à pesquisa pública ou privada	38,6 ±8,8	43 (30; 46)	20 - 46	
Organizações não assistenciais vinculadas à saúde (Ministério da Saúde, OPS^††^, ONGs^‡‡^, etc)	33,5 ±10,3	34,5 (25,5; 41,5)	10 - 50	
Trabalhar como professor em nível universitário ou técnico	35,9 ±9,4	35 (27; 44)	19 - 50	
Outros	30,3 ±12,6	29 (20; 40)	10 - 50	

*IT-PNA = Intenção de trabalhar no primeiro nível de atenção; ^†^DP = Desvio-padrão; ^‡^RIC = Intervalo interquartílico; ^§^Intervalo = Valor mínimo e máximo; ^∥^Resultado do teste U de Mann-Whitney; ^¶^Resultado do teste H de Kruskal-Wallis; **PNA = Primeiro nível de atenção; ^††^OPS = Organização Panamericana de Saúde; ^‡‡^ONGs = Organizações não governamentais

Quanto à identificação dos fatores associados à pontuação de IT-PNA, constatou-se que, das variáveis independentes, apenas a PTE-PNA é significativamente associada, aumentando 0,46 (IC 95%: 0,39; 0,53) pontos da intenção de trabalho mediana à medida que a pontuação PTE-PNA aumenta (Tabela 5).

Quanto às covariáveis estudadas, foi encontrada uma tendência negativa na pontuação IT-PNA, onde estudantes do sexo masculino tiveram 2,51 (IC 95%: -4,63; -0,39) pontos a menos na mediana IT-PNA em comparação com estudantes do sexo feminino; por outro lado, estudantes de universidades privadas tiveram 2,16 (IC 95%: -3,92; -0,40) pontos a menos na mediana IT-PNA em comparação com estudantes de universidades públicas, enquanto aqueles que não planejavam estudar especialização tiveram 7,48 (IC 95%: -13,81; -1,15) pontos a menos na mediana da IT-PNA em comparação com aqueles que ainda não haviam decidido por tais estudos. As covariáveis que favoreceram a pontuação IT-PNA foram a prontidão para o trabalho no PNA, no qual aqueles que se consideravam preparados (1,82; IC 95%: 0,02; 3.62) e bastante preparado (4.44; IC 95%: 0,67; 8.21) apresentaram uma mediana maior em comparação com aqueles que indicaram possuir treinamento regular; da mesma forma, estudantes que planejaram estudos de especialização tiveram 3,20 (IC 95%: 0,65; 5,75) pontos a mais em termos de IT-PNA em comparação com ainda não ter tomado uma decisão para tais estudos (Tabela 5).

**Tabela 5:** Fatores associados à pontuação IT-PNA* de estudantes de enfermagem de universidades do Peru, 2023-2024 (n = 740)

Características	Coef ajust^†^ (IC95%^‡^)	Valor P
Pontuações PTE-PNA^§^	0,46 (0,39; 0,53)	<0,001
Pontuações C-APS^∥^	-0,02 (-0,22; 0,19)	0,866
Sociodemográficas		
Sexo		
Feminino	Referência	
Masculino	-2,51 (-4,63; -0,39)	0,020
Estado civil		
Solteiro	Referência	
Casado	1,46 (-2,87; 5,79)	0,507
Em união estável	-0,62 (-4,42; 3,18)	0,749
Divorciado	-7,18 (-15,13; 0,77)	0,077
Outros	8,30 (-12,91; 29,5)	0,443
Ter filhos		
Não	Referência	
Sim	0,00 (-2,65; 2,64)	0,998
Dependência econômica		
Não	Referência	
Sim	0,54 (-2,00; 3,07)	0,677
Acadêmicas		
Tipo de universidade		
Público	Referência	
Privado	-2,16 (-3,92; -0,40)	0,016
Ano de estudo		
Terceiro	Referência	
Quarto	-0,44 (-3,07; 2,19)	0,742
Quinto	0,00 (-2,69; 2,69)	1,000
Graduado (ex-aluno	-3,60 (-7,28; 0,09)	0,056
Pensou em mudar de carreira		
Não	Referência	
Sim	-0,88 (-2,62; 0,86)	0,322
Estudos anteriores		
Não	Referência	
Sim	-0,71 (-2,96; 1,55)	0,538
Frequência de prácticas no PNA^¶^		
Não houve ou foram muito poucas	0,23 (-2,29; 2,74)	0,858
Poucas práticas	-0,59 (-2,77; 1,59)	0,594
Práticas moderadamente frequentes	Referência	
Práticas frequentes	-0,97 (-3,11; 1,18)	0,377
Práticas muito frequentes	0,40 (-2,47; 3,27)	0,785
Preparação para o trabalho no PNA^¶^		
Não estou preparada	-4,71 (-11,06; 1,63)	0,145
Algo preparada	-0,94 (-3,39; 1,50)	0,449
Regularmente preparada	Referência	
Estou preparada	1,82 (0,02; 3,62)	0,048
Estou bastante preparada	4,44 (0,67; 8,21)	0,021
Planeja realizar estudos de especialização		
Não	-7,48 (-13,81; -1,15)	0,021
Ainda não decidi	Referência	
Sim	3,20 (0,65; 5,75)	0,014
Planeja cursar o mestrado		
Nâo	-0,55 (-6,20; 5,10)	0,848
Ainda não decidi	Referência	
Sim	0,40 (-1,81; 2,61)	0,723
Planeja cursar o doutorado		
Não	-2,29 (-6,61; 2,04)	0,299
Ainda não decidi	Referência	
Sim	-0,76 (-2,55; 1,04)	0,409
Situação profissional		
Possui trabalho remunerado		
Não	Referência	
Sim	0,05 (-2,57; 2,67)	0,970

*IT-PNA = Intenção de trabalhar no primeiro nível de atenção laboral no primeiro nível de atenção; ^†^Coef Ajust = Coeficiente ajustado para regressão quantílica em relação à mediana; ^‡^IC95% = Intervalo de confiança de 95%; ^§^PTE-PNA = Percepção do trabalho da enfermeira no primeiro nível de atenção; ^∥^C-APS = Conhecimentos sobre atenção primária à saúde; ^¶^PNA = Primeiro nível de atenção

## Discussão

Os enfermeiros desempenham um papel fundamental no PNA, sendo os únicos profissionais de saúde disponíveis em muitos territórios e instalações de saúde do PNA, principalmente em áreas remotas ou rurais^9^ ^,^ ^27^. No entanto, a escassez de enfermeiros é um problema crítico, com tendência a se agravar devido à potencial desistência de enfermeiros do PNA e à evasão desse setor como área de desenvolvimento profissional.

O estudo mostra uma notória IT-PNA entre os estudantes, revelando uma disposição favorável ao PNA, que se baseia em crenças normativas positivas de avaliação do PNA e da AP, como a identificação de professores, referentes às políticas da APS como motivadores e à autopercepção de prontidão para atuar no PNA. Essas descobertas refletem avanços no reconhecimento do PNA como área de desenvolvimento profissional e da APS como eixo do cuidado abrangente. Embora a literatura recente na América Latina seja escassa, os resultados são consistentes com um estudo realizado no Peru que identificou uma maior disposição dos estudantes de enfermagem para trabalhar em áreas rurais em comparação com estudantes de medicina^28^. Pelo contrário, estudos no México (1,9%)^18^ ^)^ e no Peru (12,5%)^19^ ^)^ relataram IT-PNA muito baixo.

As percepções sobre o trabalho dos enfermeiros no PNA eram, em sua maioria, desfavoráveis, destacando avaliações negativas de prestígio, condições de trabalho, papel profissional no PNA, oportunidades de desenvolvimento, liderança e remuneração; esses resultados são consistentes com um estudo realizado no Brasil^29^ que revelou tensões entre o ideal da APS (ligação, território, integralidade) e limitações estruturais. Reconheceu-se a complexidade do papel da enfermagem no PNA/AP, que combina atividades de cuidado, gestão e promoção da saúde, onde a construção de vínculos com os usuários, o conhecimento do território, o trabalho com a comunidade e a abordagem intercultural são competências centrais; e que a sobrecarga administrativa, a escassez de recursos e as dificuldades no trabalho interdisciplinar limitam o pleno desenvolvimento da prática de enfermagem nesse nível^29^.

As percepções sobre o trabalho dos enfermeiros no PNA eram, em sua maioria, desfavoráveis, destacando avaliações negativas de prestígio, condições de trabalho, papel profissional no PNA, oportunidades de desenvolvimento, liderança e remuneração. Esses resultados são consistentes com um estudo realizado no Brasil(29) que revelou tensões entre o ideal da APS (ligação, território, integralidade) e limitações estruturais. Reconheceu-se a complexidade do papel da enfermagem no PNA/AP por meio da combinação de atividades de cuidado, gestão e promoção da saúde. A construção de vínculos com os usuários, o conhecimento do território, o trabalho com a comunidade e a abordagem intercultural são competências centrais. Além disso, a sobrecarga administrativa, a escassez de recursos e as dificuldades no trabalho interdisciplinar limitam o pleno desenvolvimento da prática de enfermagem nesse nível^29^.

Quanto ao conhecimento sobre APS, predominou um nível moderado, consistente com outro estudo no Peru^30^ em profissionais de saúde. A medição avaliou aspectos básicos da APS (valores, princípios, elementos) e a importância dos sistemas de saúde baseados na APS^31^. Esses resultados exigem uma integração mais robusta da APS na formação em enfermagem. Por outro lado, a relação inversa não significativa entre o conhecimento e a IT-PNA encontrada e as limitadas evidências latino-americanas a esse respeito levantam a necessidade de sua exploração em outros países. Essa avaliação do conhecimento dos estudantes prestes a se formar permitiria ajustar os currículos de treinamento em enfermagem. Nesse contexto, o instrumento projetado e validado para o Peru constitui uma base valiosa para futuras adaptações e otimizações em outros ambientes.

O estudo mostrou que percepções positivas sobre o trabalho de enfermagem no PNA e a autopercepção da preparação estão associadas a uma maior intenção de carreira no PNA. O fato de ser do sexo masculino e estudante de universidade privada tem uma influência negativa. Esses achados destacam a urgência de fortalecer o processo de treinamento na APS. Não foram encontrados antecedentes nos alunos; um estudo realizado no Brasil com enfermeiros da AP observou que percepções favoráveis do ambiente estavam associadas à maior retenção e satisfação no emprego^32^. Esses resultados sugerem que melhorar a percepção do estudante sobre o papel de enfermagem no PNA pode favorecer a atração nesse nível, sendo essa percepção um possível preditor precoce da escolha do emprego, contra o qual deve agir durante o treinamento. Essas percepções são dinâmicas e moldadas por experiências, ambientes institucionais, modelos a seguir e interação com professores e tutores, o que representa desafios significativos para a formação^33^ ^-^ ^34^ e a necessidade de reflexão nas instituições de formação.

Dada a escassez de enfermeiros na América Latina, promover a vocação para o PNA/AP é essencial para reduzir lacunas, avançar rumo a sistemas de saúde equitativos e à CUS. Universidades e cenários de prática são fundamentais para formar estudantes competentes, com percepções e atitudes positivas em relação ao PNA/APS. Nesse sentido, necessita-se urgentemente de reformas e ações estratégicas nas universidades para consolidar a preparação de enfermagem com foco no PNA/APS^33^ ^-^ ^34^, por meio da formação de profissionais íntegros e humanistas com competências^31^ e ferramentas que lhes permitam abordar as comunidades, responder às diversidades, aos determinantes sociais e às necessidades das comunidades.

Os países buscam consolidar suas estruturas de saúde com base na APS, e as universidades devem responder e contribuir para esse fim. Portanto, as reformas implicam redesenhar os currículos com foco explícito na APS/PNA^35^, ir além do modelo biomédico^34^, reconceituar e transformar o significado das práticas de enfermagem na comunidade^36^, promover práticas precoces e prolongadas em comunidades e áreas rurais, fortalecer a função de treinamento e ensino e o acompanhamento de tutores^37^, inovar estratégias de educação interprofissional, simulação^38^ ^-^ ^39^ e avaliação das percepções dos alunos. Evidências mostram que programas educacionais com experiência prática aprimoram competências^34^, aprimoram a avaliação do papel da enfermagem na AP e estimulam o interesse pelo trabalho. No entanto, ainda há limitações nos currículos, com pouca presença comunitária e ausência de referências profissionais, o que restringe a compreensão do campo de atuação da enfermagem na AP^34^.

Os achados fornecem evidências úteis para orientar políticas trabalhistas que favoreçam a atração e retenção de enfermeiros no PNA. Devido ao seu alto impacto na “percepção”, necessita-se melhorar as condições de trabalho desfavoráveis e as insatisfações percebidas no PNA/AP e em ambientes comunitários e rurais^6^ ^,^ ^9^ ^,^ ^14^ ^,^ ^29^ ^,^ ^40^ ^-^ ^41^. Definitivamente, em cada país e dentro dele, as realidades diferem e têm suas próprias peculiaridades que precisam ser analisadas. Uma governança eficaz dos sistemas de saúde é essencial para fortalecer o PNA e a APS^42^, com maior investimento em recursos e orçamento para o PNA, juntamente com políticas que possam adequar de forma abrangente as condições estruturais e de trabalho da enfermagem.

Por outro lado, para aprimorar as “percepções”, é necessário abordar a sobrecarga administrativa e de cuidados^27^, garantir infraestrutura adequada, disponibilidade de suprimentos, treinamento de pós-graduação, protocolos de cuidado^43^, maiores margens de autonomia na gestão do cuidado^44^ ^-^ ^45^ e na gestão de equipes^46^. Promover o reconhecimento social e organizacional do papel dos enfermeiros^13^, bem como fornecer apoio psicossocial que favoreça seu bem-estar. No âmbito econômico, é necessário garantir contratos estáveis, remuneração justa^13^ ^-^ ^14^ e estabelecer incentivos diferenciados (escalas salariais, bônus, benefícios como moradia, transporte, alimentação, entre outros) para atrair e manter enfermeiros no PNA, especialmente em áreas rurais, amazônicas e de grande altitude, o que permitirá a continuidade e qualidade em um atendimento abrangente de saúde nessas áreas.

Percepções negativas sobre o papel da enfermagem no PNA refletem desvalorização e pouco reconhecimento profissional, o que coincide com uma valorização social historicamente desfavorável da enfermagem^6^ ^,^ ^9^ ^,^ ^14^ ^,^ ^40^ ^,^ ^47^. Tais questões exigem que organizações de enfermagem, universidades, autoridades e os próprios enfermeiros promovam ações para reavaliar e tornar visível^48^ o impacto de seu trabalho, reforçando sua identidade e compromisso profissional^49^. O PNA, devido à sua complexidade e relevância na liderança de programas com impacto na saúde das famílias e comunidades^12^, é um espaço estratégico para o posicionamento e empoderamento da enfermagem^50^. No entanto, exige melhorias sustentadas nas condições de trabalho e nos salários para quem trabalha nesse nível.

Este trabalho é o primeiro com uma abordagem quantitativa realizada com estudantes peruanos. Recomenda-se desenvolver estudos longitudinais multicêntricos, incluindo métodos qualitativos para maior compreensão dos facilitadores e obstáculos das preferências de carreira dos estudantes em diversos contextos. Suas principais limitações são: a) As universidades foram selecionadas por conveniência, o que poderia introduzir viés de seleção e limitar sua representatividade; b) O desenho foi um estudo transversal, portanto não estabelece relações causais; e c) É provável que a experiência e o conhecimento limitados dos estudantes sobre o papel real dos enfermeiros no PNA possam ter influenciado sua percepção ou que eles tenham ajustado suas respostas devido a uma questão de desejabilidade social. Portanto, os resultados devem ser interpretados com cautela, levando-se em consideração tais desfechos.

## Conclusão

Percepções favoráveis do trabalho de enfermagem no PNA têm um impacto positivo na intenção de emprego dos estudantes de enfermagem nesse nível. Por outro lado, o conhecimento sobre APS não influenciaria a intenção de trabalho. No entanto, estudos adicionais são recomendados, considerando que a análise bivariada sugeriu que, quanto maior o conhecimento sobre a APS, menor a intenção de atuar no PNA.

A autopercepção de estar preparado para trabalhar no PNA tem uma influência favorável no interesse pelo desenvolvimento de empregos nesse nível, ao contrário do que acontece com estudantes de universidades privadas e do sexo masculino. A predominância de estudantes com nível moderado de conhecimento sobre a APS exige uma integração mais robusta da APS na educação em enfermagem.

Os achados revelam a importância de abordar a percepção da imagem e do papel da enfermagem no PNA durante a educação universitária, de formular julgamentos e de adquirir uma visão adequada da enfermagem que favoreça uma carreira no PNA. Além disso, reverter as percepções desfavoráveis encontradas e superar a alta proporção de estudantes com nível médio de conhecimento sobre a APS.

Por outro lado, é necessário promover estratégias para a reavaliação, visibilidade e empoderamento da enfermagem no PNA, bem como para a defesa da melhoria das condições de trabalho no PNA e do fortalecimento do PNA, que permitam reverter a realidade adversa que enfrenta, tornando o trabalho nesse nível mais atraente.

## Data Availability

Os conjuntos de dados relacionados a este artigo estarão disponíveis mediante solicitação ao autor correspondente.

# Translation: Percepciones y conocimientos en atención primaria de salud e intención laboral en el primer nivel de atención: estudio transversal^*^

**DOI:** 10.1590/1518-8345.8021.4972

**Authors:** Maricela Curisinche-Rojas, Tula Margarita Espinoza-Moreno, Reyna Ysmelia Peralta-Gómez, Lucy Zenelia Barrantes-Cruz, Gilmer Solis-Sánchez, Irma Yupanqui-Martinez, Lida Inés Carhuas-Peña, Cindy Vargas-Cabrera, Juvita Dina Soto-Hilario, Tomás Caycho-Rodriguez

**Affiliations:** 1 Universidad Científica del Sur, Facultad de Enfermería, Lima, Peru.; 2 Universidad Nacional Mayor de San Marcos, Lima, Peru.; 3 Universidad Nacional de San Agustín, Departamento Académico de Enfermería, Arequipa, Peru.; 4 Universidad Privada Antenor Orrego, Trujillo, La Libertad, Peru.; 5 Universidad Científica del Sur, Lima, Peru.; 6 Universidad Nacional Daniel Alcides Carrión, Facultad de Ciencias de la Salud-Escuela de Formación Profesional de Enfermería, Cerro de Pasco, Pasco, Peru.; 7 Universidad Nacional de Huancavelica, Facultad de Enfermería, Huancavelica, Peru.; 8 Universidad Señor de Sipán, Escuela de Enfermería, Chiclayo, Lambayeque, Peru.; 9 Universidad Nacional Hermilio Valdizán, Huánuco, Peru.

**Keywords:** Enfermería, Atención Primaria de Salud, Percepción, Conocimiento, Intención, Trabajo.

## Abstract

**Objetivo:**

determinar asociación de la percepción del trabajo de enfermería y conocimiento sobre atención primaria de salud con intención laboral en el primer nivel de atención.

**Método:**

estudio transversal analítico, realizado en 740 estudiantes universitarios de enfermería. Se valoraron intención laboral en el primer nivel de atención, percepción del trabajo de enfermería y conocimientos sobre atención primaria de salud usando instrumentos ad-hoc validados. Se realizó análisis bivariado y de regresión de cuantiles a la mediana.

**Resultados:**

predominaron alta intención laboral (66,8%), percepción del trabajo de enfermería poco favorable (53,0%) y nivel moderado de conocimientos (67,0%). La intención laboral se asoció positivamente con la percepción del trabajo (Coeficiente ajustado: 0,46, IC95%: 0,39; 0,53), con la autopercepción de preparación para el trabajo y con tener planificada una especialización. El sexo masculino y estudiar en universidades privadas se asociaron negativamente. No se encontró asociación significativa con conocimientos.

**Conclusión:**

percepciones favorables del trabajo de enfermería en el primer nivel de atención aumentan la intención laboral en este nivel, mientras que el conocimiento sobre atención primaria de salud no tendría influencia. Abordar la percepción del trabajo durante la formación universitaria es fundamental para favorecer el interés laboral de los futuros enfermeros.

## Destacados

**(1)** Futuros enfermeros en la atención primaria ayudarán a reducir brechas en enfermería.

**(2)** La percepción favorable de enfermería eleva la intención laboral en atención primaria.

**(3)** Predominó el nivel medio de conocimientos sobre atención primaria de salud.

**(4)** En universidad privada y hombres es menor la intención laboral en atención primaria.

**(5)** La academia debe forjar visión positiva de enfermería en el primer nivel de atención.

## Introducción

“No dejar a nadie atrás” y alcanzar la cobertura universal de salud (CUS) en el mundo se vislumbran cada vez más lejanos. Enfrentamos un preocupante panorama global de estancamiento en la CUS con más del 50% de la población mundial sin plena cobertura de servicios esenciales de salud y muchos hogares afrontando gastos sanitarios catastróficos^1^. En países latinoamericanos, persisten índices elevados de necesidades de atención de salud insatisfechos y presencia de diversas barreras de acceso que limitan el desempeño de la atención primaria de salud (APS)^2^ ^-^ ^3^. Ahora, más que nunca, urge consolidar los sistemas de salud con un enfoque en la APS como eje central de su organización y gestión. La APS es la estrategia más inclusiva, pro-equidad, costo-efectiva y eficiente para enfrentar barreras de acceso, aumentar la protección financiera en los hogares y avanzar hacia los objetivos de desarrollo sostenibles^1^ ^-^ ^2^. En Perú, son evidentes los esfuerzos y avances por posicionar la APS como cimiento del sistema de salud; sin embargo, pasar de un modelo conceptual a una implementación auténtica aún es un proceso lento^4^.

El primer nivel de atención (PNA) es fundamental para la implementación efectiva de los atributos de la APS (primer contacto, longitudinalidad, integralidad y coordinación). Constituye la puerta de entrada y base de la pirámide sanitaria, con amplia presencia en comunidades rurales y urbanomarginales, se integra y articula con otros niveles de atención para garantizar la continuidad del cuidado. Este nivel brinda atención primaria (AP) que comprende el cuidado integral de la salud por curso de vida para la persona, familia y comunidad, la promoción de la salud y prevención de riesgos y daños en corresponsabilidad con la comunidad organizada y el gobierno local para el abordaje de los determinantes sociales de la salud^5^. Sin embargo, el PNA enfrenta serios problemas que limitan su desempeño^2^ ^,^ ^6^, siendo la escasez de enfermeras un determinante crítico^7^ ^-^ ^8^.

Las enfermeras son la principal fuerza laboral en salud y cumplen un papel primordial en el PNA y en la APS^8^, principalmente en zonas rurales y de menor desarrollo^9^. Dado su amplio perfil profesional y visión salutogénica son especialistas en el cuidado integral de la salud de la persona, familia y comunidad^10^. El ejercicio de la enfermería como esencia propia de la APS^7^, su liderazgo y autonomía en el cuidado de la salud y, su responsabilidad social, ética y humanística con la APS, están ampliamente reportados a nivel global^7^ ^,^ ^11^ y en América Latina^9^ ^,^ ^12^. No obstante, en Latinoamérica los enfermeros del PNA enfrentan condiciones de trabajo desfavorables que limitan el ejercicio de la AP y tienen influencia negativa en la satisfacción laboral e inclinaciones de permanencia en el PNA^6^ ^,^ ^9^ ^,^ ^13^ ^-^ ^14^; similar a lo observado en otras regiones^15^. Destacan escasez y distribución inequitativa de enfermeros, sobrecarga de trabajo y administrativa, turnos extensos, exiguos recursos, infraestructura inadecuada, estrés laboral, insuficiente reconocimiento, precarización laboral, entre otros^6^ ^,^ ^9^ ^,^ ^13^ ^-^ ^14^, y esta coyuntura no es ajena a la realidad peruana^16^. La ausencia o rotación de los enfermeros en el PNA afecta el acceso a la AP^17^ y la atracción y retención laboral de estos profesionales en el PNA son fundamentales. De hecho, la evidencia reporta que estás se vinculan a óptimas condiciones estructurales en la práctica, percepciones favorables y bienestar laboral^15^. 

Los estudiantes representan la reserva estratégica futura de la enfermería que contribuirá a cerrar las brechas en AP. Por tanto, es prioritario prestar atención a sus expectativas e intención de trayectoria laboral y explorar factores que los facilitan o limitan. En Latinoamérica, antecedentes al respecto son escasos. Dos estudios en México y Perú advirtieron escasas expectativas de trabajo en el PNA y preferencias por el campo hospitalario^18^ ^-^ ^19^; sin embargo, no profundizaron el análisis de factores o explicaciones de esta tendencia, lo cual subraya la necesidad de estudios al respecto. 

La evidencia en otros territorios del ámbito global advierte bajas preferencias laborales en escenarios de AP por parte de estudiantes de enfermería^20^ ^-^ ^23^ y que percepciones inadecuadas sobre la APS y el rol de enfermería, respecto a prestigio, salario y condiciones laborales, actúan como barreras para su elección del PNA/AP como campo profesional. En contraste, la procedencia rural, la autonomía y una adecuada exposición curricular se reconocen como factores facilitadores^20^ ^-^ ^23^. En Perú se desconoce que está ocurriendo sobre el particular.

En este marco, el estudio parte de la premisa de que los estudiantes de enfermería peruanos próximos o recientemente egresados que tienen percepciones favorables sobre el rol de enfermería en el PNA y aquéllos, que tienen adecuados conocimientos sobre APS adquiridos durante su formación profesional tendrían una mayor intención de trabajo en el PNA. En este sentido, la pregunta que orientó la investigación fue: ¿La percepción del trabajo de la enfermera en el PNA y el conocimiento sobre APS están asociados con la intención laboral en el PNA de estudiantes de enfermería de universidades peruanas?

El objetivo del estudio fue determinar la asociación entre la percepción del trabajo de la enfermera en el PNA y el conocimiento sobre APS con la intención laboral en el PNA de estudiantes de enfermería del Perú. La evidencia generada servirá de apoyo en la toma de decisiones para mejorar planes de estudio y procesos de formación; planificar y alinear la oferta formativa con las demandas reales del sistema de salud; desarrollar programas de motivación e incentivos para atraer y retener talento joven en el PNA y proponer políticas de fortalecimiento del PNA. 

## Método

### Diseño del estudio 

Se realizó un estudio observacional, transversal, analítico siguiendo las directrices del *Strengthening the Reporting of Observational Studies in Epidemiology* (STROBE). 

### Lugar y período de recolección de datos

El estudio se llevó a cabo en 11 universidades públicas y privadas del Perú que ofrecen programas de enfermería, ubicadas en cuatro macrozonas del país: norte, centro, sur y Lima Metropolitana. Esta distribución permitió contar con representantes de territorios con diversas realidades sociales, formativas y sanitarias. 

La viabilidad operativa del estudio (autorización institucional y contar con un coinvestigador de la universidad) y el tamaño de la población estudiantil fueron criterios para seleccionar las universidades.

En el norte se incluyeron dos universidades ubicadas en ciudades costeras de alta concentración urbana (Trujillo y Chiclayo), con presencia de servicios de salud en contextos urbanos y rurales. En la región centro, fueron incluidas tres universidades de zonas andinas (Huancavelica, Huánuco y Cerro de Pasco), caracterizadas por el predominio de poblaciones rurales y dispersas, mayor pobreza y presencia prioritaria de centros de salud del PNA, mientras que en el sur, fue considerada una universidad en Arequipa, segunda ciudad del país y polo regional que cuenta con servicios de alta especialización y del primer nivel de atención. Finalmente, en Lima Metropolitana, capital del país y principal centro académico y laboral en salud, caracterizada por su alta concentración estudiantil y diversidad institucional, fueron seleccionadas cinco universidades.

Se seleccionaron universidades públicas y privadas considerando que ambas representan la diversidad existente en la educación superior en enfermería, con diferencias en condiciones de formación académica y en las perspectivas de acceso al mercado laboral según el tipo de gestión institucional.

La recolección de datos se realizó entre noviembre de 2023 y febrero de 2024.

### Población, criterios de selección y muestra

La población estuvo compuesta por estudiantes de enfermería matriculados entre el tercer y quinto año (último año) y egresados del año 2022 de las universidades seleccionadas. Se priorizó a estudiantes de ciclos avanzados por su mayor exposición curricular teórico-práctico sobre APS/PNA, y a egresados recientes, para integrar la perspectiva de transición al mercado laboral. 

No se estimó tamaño de muestra, participaron todos los estudiantes que cumplían los criterios de elegibilidad que fueron tener 18 años a más y que otorgaron consentimiento informado. Se excluyeron estudiantes con retiro temporal o ausentes en las fechas de la encuesta.

### Variables

Las principales variables fueron la intención laboral en el primer nivel de atención (IL-PNA), la percepción de trabajo de enfermería en el primer nivel de atención (PTE-PNA) y el conocimiento sobre atención primaria de salud (C-APS) las cuales se midieron de manera numérica a través de puntuaciones obtenidas de escalas tipo Likert. Se midió la PTE-PNA y la IL-PNA de manera general y por cada una de sus dimensiones; adicionalmente se categorizó la distribución de los puntajes obtenidos en tres niveles mediante intervalos de clase.

Se recolectó información de otras covariables según características de los encuestados: i) Sociodemográficas, ii) Académicas y iii) Laborales. A nivel sociodemográfico se registró la edad, sexo, estado civil, tener hijos (si/no), y dependencia económica; mientras que las características académicas evaluadas fueron el tipo de universidad, la zona del país donde se ubica la universidad, año de estudio, si pensó cambiarse de carrera, contaba con estudios previos (a nivel técnico o universitario), frecuencia de prácticas en el PNA, preparación para trabajo en el PNA, planes para cursar especialización, maestría, y/o doctorado. A nivel laboral se registró si tenían trabajo remunerado, sector y lugar de trabajo y dónde planeaban trabajar. 

### Instrumentos de recolección de datos

La PTE-PNA, IL-PNA, y el C-APS fueron medidas a través de escalas ad-hoc construidas específicamente para este estudio. Las escalas para medir la PTE-PNA e IL-PNA constaban de declaraciones o ítems con cinco alternativas de respuestas tipo Likert: No estoy de acuerdo, Algo de acuerdo, Regular de acuerdo, Estoy de acuerdo, Totalmente de acuerdo; las cuales se puntuaban de 1 a 5, respectivamente. Por su parte, la escala para medir el C-APS estuvo conformada por afirmaciones de opción verdadero falso, asignándole puntaje 0 cuando la respuesta era incorrecta y 1 cuando era correcta.

La escala propuesta para la PTE-PNA constaba de 13 declaraciones distribuidas en tres dimensiones: 1 - Imagen de la enfermera que trabaja en el PNA, 2 - Trabajo en el PNA, y 3 - Consecuencias económicas de trabajar en el PNA. Por su parte, la escala para la medición de IL-PNA constaba de 10 declaraciones distribuidas en tres dimensiones: 1 - Actitud para laborar en el PNA, 2 - Creencias normativas subjetivas y 3 - Creencias o auto percepción de la capacidad de control del comportamiento. La escala de C-APS contó con 31 afirmaciones, sin distribución en dimensiones. Adicionalmente, se utilizó un cuestionario específico para las características sociodemográficas, académicas, y laborales.

### Validación de los instrumentos de recolección de datos

La elaboración de la escala de IL-PNA se enmarcó en la Teoría del Comportamiento Planificado^24^ ^-^ ^25^ y las declaraciones por cada dimensión fueron formuladas a partir de revisión bibliográfica. Respecto a la escala de C-APS, se consideraron afirmaciones sobre los valores, principios y elementos de la APS e importancia del sistema de salud basado en APS identificados a través de la revisión de literatura científica y documentos técnicos. Por su parte, la escala de PTE-PNA resultó de la reestructuración y adecuación al contexto de enfermería de una escala usada en estudiantes de medicina en Latinoamérica^26^.

Las tres escalas fueron validadas por 11 jueces expertos integrados por enfermeras especialistas, con maestría y/o doctorado en salud pública, comunitaria y/o familiar; con experiencia mayor a 10 años laborando en el PNA y/o en gestión de programas de salud en el nivel regional o nacional, procedentes de Lima y de las regiones involucradas en el estudio. 

A través de formulario online compartido, los jueces desarrollaron las matrices de validación de los ítems de cada escala, considerando criterios de relevancia, claridad y pertinencia. Los resultados fueron analizados mediante el coeficiente V de Aiken obteniéndose grados de acuerdo aceptables en todas las declaraciones (ítems), para la PTE-PNA entre 0,75 y 1,00, para la IL-PNA entre 0,73 y 0,97 y para el C-APS entre 0,73 y 1,00. 

Se determinó la confiabilidad de los instrumentos a través de la consistencia interna mediante el coeficiente Omega de McDonald. Para cada dimensión de la PTE-PNA la confiabilidad fue de 0,908 (D1), 0,827 (D2) y 0,834 (D3); mientras que para las dimensiones de la IL-PNA estos fueron de 0,855 (D1), 0,914 (D2) y 0,880 (D3). Finalmente, para el C-APS este coeficiente fue de 0,794.

Debida a la distribución de las afirmaciones de la PTE-PNA e IL-PNA en dimensiones, se realizó la validez de constructo mediante análisis factorial confirmatorio, encontrando índices de ajuste aceptables para ambas escalas (p<0,001). Específicamente para la PTE-PNA se encontró un *Comparative Fit Index (*CFI) de 0,963, un *Root Mean Square Error of Approximation (*RMSEA) de 0,080 (Intervalo de confianza al 95% [IC95%]: 0,070; 0,102), y un *Standardized Root Mean Square Residual (*SRMR) de 0,03. En cuanto a la escala de IL-PNA, el CFI fue de 0,950, el RMSEA fue 0,117 (IC95%: 0,103; 0,131) y el SRMR fue 0,03.

### Recopilación de datos

Antes de iniciar la recolección de datos, se llevaron a cabo reuniones presenciales y virtuales para coordinar y organizar las actividades del estudio. Asimismo, mediante sesiones virtuales se capacitó a los responsables de la recopilación en los procedimientos de obtención del consentimiento informado y aplicación de los instrumentos.

La recolección de datos fue realizada por coinvestigadores docentes de cada universidad, quienes gestionaron las autorizaciones, coordinaron las fechas y reunieron a los participantes en espacios institucionales. Tras verificar los criterios de elegibilidad, se aplicaron los instrumentos en forma anónima, sin registro de nombres o apellidos.

Para el caso de los egresados del año 2022 y estudiantes que no acudían a las reuniones presenciales se utilizó una versión digital de los instrumentos en Google *Forms* que fue compartido mediante correo electrónico o vía WhatsApp, para luego, proceder a realizar seguimiento, con envío de recordatorios a fin de maximizar la tasa de respuesta.

### Tratamiento y análisis de datos

Los datos recolectados fueron organizados en una hoja de cálculo de MS Excel y luego analizados mediante estadística descriptiva e inferencial usando STATA v. 17.0 (Stata Corporation, College Station, Texas, USA) con la cual se estimaron medidas de frecuencia absoluta (n) y relativa (%) de las variables cualitativas recolectadas. Se identificó la distribución de frecuencia absoluta y relativa de las respuestas de cada pregunta de los tres (03) cuestionarios aplicados.

Lo puntajes obtenidos de la sumatoria de cada dimensión y de la escala en total para cada uno de los cuestionarios fueron evaluados mediante medidas de tendencia central (media y mediana) y dispersión (rango, desviación estándar, rango intercuartil). A partir de los puntajes de cada escala se generaron tres intervalos de clase mediante regla de Sturges categorizados en Desfavorable, Poco favorable y Favorable para el PTE-PNA; mientras que para la IL-PNA y el C-APS fue en Bajo, Moderado y Alto. Estos intervalos fueron determinados de manera general y por cada dimensión de la escala

Se evaluó la diferencia del puntaje del cuestionario de intención laboral para cada una de sus categorías y en la escala total según las categorías de los intervalos de clase de las dimensiones y escala total de los cuestionarios de conocimiento en APS y el de percepción del trabajo de la enfermera en el PNA, así también según las categorías de las covariables de estudio. Esta comparación se realizó mediante la prueba T de Student para muestras independientes o U de Mann-Whitney y análisis de varianza de un factor (ANOVA) o H de Kruskal-Wallis. La elección de las pruebas se basó en la identificación de distribución normal o no normal (prueba de Shapiro-Wilk) y en la cantidad de grupos a comparar (2 o más de 3). 

Se identificó la relación entre el puntaje del cuestionario de intención laboral con los puntajes de los cuestionarios de conocimiento en atención primaria de salud y el de percepción del trabajo de la enfermera en el PNA mediante las pruebas de coeficiente de correlación de Pearson o Spearman según de distribución normal o no normal (prueba de Shapiro-Wilk). Para la evaluación de los factores asociados al puntaje del cuestionario de intención laboral se realizó una regresión de cuantiles a la mediana ajustada, considerando como variables de ajuste a las variables independientes (puntaje de los cuestionarios de conocimiento en atención primaria de salud y el de percepción del trabajo de la enfermera en el PNA) y las covariables que tuvieron un valor de p menor de 0,20 en el modelo crudo; se consideraron varianzas robustas a fin de evitar afectación del modelo por errores por heterocedasticidad en el modelo. Todos los resultados se trabajaron con un nivel de significancia de 0,05.

### Aspectos éticos

Antes de iniciar la ejecución del estudio, el proyecto de investigación fue aprobado por el Comité Institucional de Ética en Investigación de la Universidad Científica del Sur (Constancia N°055-CIEI-Científica-2023) y se obtuvieron la autorización de las universidades participantes. Previo a la aplicación de los instrumentos se brindó información detallada del estudio y se obtuvo el consentimiento informado firmado de los estudiantes que aceptaron participar del estudio y para el caso de la recolección de datos virtual, el instrumento digital (Google *Forms)* contaba con una primera sección con explicación minuciosa del estudio, sobre aspectos relacionados a la participación del estudiante en el estudio y un consentimiento electrónico en el cual debía elegir entre la opción de participar o no del estudio. Los estudiantes que aceptaban participar voluntariamente desarrollaron los instrumentos.

Se salvaguardó los datos personales mediante mecanismos de anonimización, siendo estos de acceso único para los investigadores.

## Resultados

De los 740 estudiantes de enfermería que participaron en el estudio, la media de la edad fue de 24,4 ±4,9 años; el 85% eran mujeres, el 89,2% no estaban casados, el 84,2% no tenían hijos, el 74,3% eran económicamente dependientes, el 76,4% no tenían empleo remunerado, el 59,5% estudiaban en universidades públicas, el 44,2% estudiaban en universidades de Lima, el 44,5% estaban en su cuarto año de estudio, el 71,9% no planeaban cambiar de carrera y el 80,0% no tenían educación superior previa. En cuanto a la formación, el 32,0% había realizado prácticas con una frecuencia moderada, el 44,3% declaró estar preparado para trabajar en el PNA, el 64,3% manifestó su intención de trabajar en un “hospital, clínica o instituto especializado”, el 87,4% planeaba cursar estudios especializados, el 77,4% tenía previsto cursar una maestría y el 53,6% tenía previsto cursar un doctorado (Tabla 1).

**Tabla 1:** Características de los estudiantes de enfermería encuestados en el Perú, 2023-2024 (n = 740)

Características	n | Media	% | DE*
Sociodemográficas		
Edad (Media ±DE*)	24,4	4,9
Sexo		
Femenino	629	85,0
Masculino	111	15,0
Estado civil		
Soltero	660	89,2
Casado	31	4,2
Conviviente	41	5,5
Divorciado	7	0,9
Otros	1	0,1
Tiene hijos		
No	623	84,2
Si	117	15,8
Dependencia económica		
No	190	25,7
Sí	550	74,3
**Académicas**		
Tipo de universidad		
Público	440	59,5
Privado	300	40,5
Zona del país donde se ubica la universidad		
Centro	178	24,1
Norte	106	14,3
Sur	129	17,4
Lima	327	44,2
Año de estudio		
Tercero	80	10,8
Cuarto	329	44,5
Quinto	264	35,7
Egresado	67	9,1
Pensó cambio de carrera		
No	532	71,9
Sí	208	28,1
Estudios previos		
No	592	80,0
Si	148	20,0
A nivel técnico	119	80,4
A nivel universitario	29	19,6
Frecuencia de prácticas en el PNA^†^		
No hubo o fueron muy escasas prácticas	103	13,9
Escasas prácticas	149	20,1
Prácticas moderadamente frecuentes	237	32,0
Prácticas frecuentes	174	23,5
Prácticas muy frecuentes	77	10,4
Preparación para trabajo en el PNA^†^		
No estoy preparada	12	1,6
Algo preparada	102	13,8
Regular preparada	256	34,6
Estoy preparada	328	44,3
Estoy muy preparada	42	5,7
Planea estudios especialización		
No	13	1,8
Aún no he decidido	80	10,8
Sí	647	87,4
Planea estudios maestría		
No	21	2,8
Aún no he decidido	146	19,7
Sí	573	77,4
Planea estudios doctorado		
No	35	4,7
Aún no he decidido	308	41,6
Sí	397	53,6
**Laborales**		
Tiene trabajo remunerado		
No	565	76,4
Sí	175	23,6
Sector donde trabaja (n=175)		
Trabajo en otros sectores	67	38,3
Trabaja en sector salud	108	61,7
Lugar de trabajo remunerado (n=175)		
Centro o puesto de salud del PNA	27	15,4
Hospital, clínica o instituto especializado	81	46,3
Otros sectores no salud	67	38,3
Dónde planea trabajar		
Hospital, Clínica, Instituto especializado	478	64,6
Centro o puesto de salud del PNA^†^	153	20,7
Instituciones dedicadas a investigación público o privado	31	4,2
Organizaciones no asistenciales vinculadas a la salud (Ministerio de Salud, OPS^‡^, ONGs^§^, etc)	40	5,4
Trabajar como profesor a nivel universitario o técnico	23	3,1
Otros	15	2,0

*DE = Desviación estándar; ^†^PNA = Primer nivel de atención; ^‡^OPS = Organización Panamericana de la Salud; ^§^ONGs = Organizaciones no Gubernamentales

Acorde a las respuestas obtenidas en los cuestionarios, en cuanto a la evaluación de la IL-PNA y para la PTE-PNA se encontró una mediana de puntajes de 39 (RIC: 30 - 39); mientras que la mediana de C-APS fue de 18 (RIC: 16 - 18). Los puntajes se clasificaron según nivel, encontrando que el 66,8% tenía una valoración alta para la IL-PNA, el 53,0% tenía una percepción poco favorable respecto del trabajo de la enfermera en el PNA y existía un 67,0% de estudiantes con un nivel de conocimiento moderado sobre la APS (Tabla 2).

La relación entre el puntaje de IL-PNA y PTE-PNA presentó una correlación estadísticamente significativa (p<0,001) con un valor de Rho de 0,531; la relación entre el puntaje de IL-PNA y C-APS evidenció una correlación estadísticamente significativa de manera inversa con un valor de Rho de -0,166 (Tabla 3).

**Tabla 2:** Puntajes y niveles obtenidos en la valoración de la IL-PNA*, PTE-PNA^†^ y C-APS^‡^ en estudiantes de enfermería del Perú, 2023-2024 (n = 740)

Variables	Puntajes			Nivel n (%)		
	Media ±DE^§^	Mediana (RIC^ǁ^)	Rango^¶^	Bajo / Desfavorable**	Moderado / Poco Favorable**	Alto / Favorable**
IL-PNA*						
Dimensión: Actitud para laborar en el PNA^††^	14,3 ±3,7	16 (12 - 16)	4 - 20	99 (13,4)	206 (27,8)	435 (58,8)
Dimensión: Creencias normativas subjetivas (presión social)	11,5 ±3,2	12 (9 - 12)	3 - 15	109 (14,7)	188 (25,4)	443 (59,9)
Dimensión: Creencias o autopercepción de la capacidad de control del comportamiento	11,2 ±3,1	12 (9 - 12)	3 - 15	105 (14,2)	239 (32,3)	396 (53,5)
Valoración general de la IL-PNA*	36,9 ±9,3	39 (30 - 39)	10 - 50	28 (3,8)	218 (29,5)	494 (66,8)
PTE-PNA^†^						
Dimensión: Imagen de la enfermera (o) que trabaja en el PNA^††^	14,4 ±5,1	15 (10 - 15)	5 - 25	250 (33,8)	276 (37,3)	214 (28,9)
Dimensión: El trabajo en el PNA^††^	19,0 ±5,3	20 (16 - 20)	6 - 30	113 (15,3)	327 (44,2)	300 (40,5)
Dimensión: Consecuencias económicas de trabajar en el PNA^††^	5,5 ±2,0	6 (4 - 6)	2 - 10	551 (74,5)	144 (19,5)	45 (6,1)
Valoración general de la PTE-PNA^†^	38,9 ±11,3	39 (30 - 39)	13 - 65	76 (10,3)	392 (53,0)	272 (36,7)
C-APS^‡^						
Valoración general del C-APS^‡^	19,0 ±3,8	18 (16 - 18)	10 - 30	0 (0,0)	496 (67,0)	244 (33,0)

*IL-PNA = Intención laboral en el primer nivel de atención; ^†^PTE-PNA = Percepción del trabajo de la enfermera en el primer nivel de atención; ^‡^C-APS = Conocimientos sobre atención primaria de salud; ^§^DE = Desviación estándar; ^∥^RIC = Rango Intercuartil; ^¶^Rango = Valor mínimo y máximo; **Valor aplicable para PTE-PNA; ^††^PNA = Primer nivel de atención

**Tabla 3:** Evaluación de la correlación del puntaje de IL-PNA* con los puntajes de PTE-PNA^†^ y de C-APS^‡^ en estudiantes de enfermería del Perú, 2023-2024 (n =740)

	Rho^§^	Valor de P
PTE-PNA^†^		
Dimensión: Imagen de la enfermera (o) que trabaja en el PNA^∥^	0,508	<0,001
Dimensión: El trabajo en el PNA^∥^	0,485	<0,001
Dimensión: Consecuencias económicas de trabajar en el PNA^∥^	0,408	<0,001
Valoración general de la PTE-PNA^†^	0,531	<0,001
C-APS^‡^		
Valoración general del C-APS^‡^	-0,166	<0,001

*IL-PNA = Intención laboral en el primer nivel de atención; ^†^PTE-PNA = Percepción del trabajo de la enfermera en el primer nivel de atención; ^‡^C-APS = Conocimientos sobre atención primaria de salud; ^§^RHO = Coeficiente Rho de Spearman; ^∥^PNA = Primer nivel de atención

Los puntajes de IL-PNA variaron según las categorías de algunas variables (p<0,05), observándose puntajes más altos entre las estudiantes mujeres, estudiantes con dependencia económica, estudiantes de universidades públicas, del tercer año, que no consideraban un cambio de carrera, los que indicaron que las prácticas en el PNA fueron muy frecuentes y que se consideraban bien preparadas para trabajar en el PNA. En cuanto a la intención de estudios futuros, tanto quienes tenían intención de cursar una especialización o una maestría presentaron puntajes más altos en la IL-PNA, mientras que, respecto a la intención de estudios de doctorado, los puntajes más altos fueron de quienes aún no habían decidido por dichos estudios (Tabla 4).

Respecto a la identificación de los factores asociados al puntaje de IL-PNA, se encontró que de las variables independientes solo la PTE-PNA se asocia significativamente incrementando en 0,46 (IC 95%: 0,39; 0,53) puntos de la mediana de intención laboral según aumenta el puntaje de PTE-PNA (Tabla 5).

**Tabla 4:** Puntajes de la IL-PNA* según características de las estudiantes de enfermería en el Perú, 2023-2024 (n = 740)

Características	Puntaje de IL-PNA*			Valor de P
	Media ±DE^†^	Mediana (RIC^‡^)	Rango^§^	
Sociodemográficas				
Sexo				
Femenino	37,3 ±9,1	40 (31; 46)	10 - 50	0,028^∥^
Masculino	34,7 ±10,4	38 (27; 43)	10 - 50	
Estado civil				
Soltero	37,2 ±9,0	39 (31; 45)	10 - 50	0,067^¶^
Casado	37,8 ±11,0	40 (27; 47)	10 - 50	
Conviviente	33,0 ±12,3	33 (21; 45)	10 - 50	
Divorciado	29,3 ±10,5	27 (26; 30)	15 - 50	
Otros	3,00 ±0,0	30 (30; 30)	30 - 30	
Tiene hijos				
No	37,2 ±9,0	40 (31; 45)	10 - 50	0,148^∥^
Si	35,3 ±10,7	39 (26; 45)	10 - 50	
Dependencia económica				
No	35,0 ±10,0	37,5 (27; 43)	10 - 50	0,002^∥^
Sí	37,6 ±9,0	40 (32; 46)	10 - 50	
Académicas				
Tipo de universidad				
Público	37,8 ±8,9	40 (32; 46)	10 - 50	0,003^∥^
Privado	35,7 ±9,8	39 (28; 43)	10 - 50	
Año de estudio				
Tercero	40,0 ±9,0	45,5 (36; 46)	14 - 50	0,001^¶^
Cuarto	36,5 ±9,5	39 (30; 44)	10 - 50	
Quinto	37,0 ±8,9	39 (31,5; 44)	10 - 50	
Egresado	35,3 ±9,7	37 (28; 43)	10 - 50	
Pensó cambio de carrera				
No	37,7 ±9,3	40 (31; 46)	10 - 50	<0,001^∥^
Sí	35,1 ±9,1	37 (28; 41)	10 - 50	
Estudios previos				
No	37,3 ±9,1	40 (31; 46)	10 - 50	0,096^∥^
Si	35,6 ±10,1	38 (27,5; 44)	10 - 50	
Estudios concluidos previos				
No	37,3 ±9,1	40 (31; 46)	10 - 50	0,249^¶^
A nivel técnico	35,5 ±10,2	38 (27; 44)	10 - 50	
A nivel universitario	35,7 ±9,7	39 (30; 41)	10 - 50	
Frecuencia de las prácticas en el PNA**				
No hubo o fueron muy escasas prácticas	36,6 ±9,4	39 (30; 45)	10 - 50	<0,001^¶^
Escasas prácticas	35,0 ±8,9	38 (28; 40)	13 - 50	
Prácticas moderadamente frecuentes	38,0 ±9,5	40 (32; 46)	10 - 50	
Prácticas frecuentes	36,4 ±9,1	39 (30; 42)	10 - 50	
Prácticas muy frecuentes	39,0 ±9,4	41 (34; 46)	14 - 50	
Preparación para trabajo en el PNA**				
No estoy preparada	31,1 ±12,0	33 (24,5; 40)	10 - 49	<0,001^¶^
Algo preparada	33,4 ±9,5	35 (25; 40)	13 - 50	
Regular preparada	35,7 ±8,9	38 (30; 42)	10 - 50	
Estoy preparada	38,8 ±8,9	40 (34; 46)	10 - 50	
Estoy muy preparada	40,5 ±9,9	44 (33; 49)	20 - 50	
Planea estudios especialización				
No	26,5 ±12,5	24 (20; 36)	10 - 48	<0,001^¶^
Aún no he decidido	33,8 ±9,2	35 (26; 40)	10 - 50	
Sí	37,5 ±9,1	40 (31; 46)	10 - 50	
Planea estudios maestría				
No	30,8 ±12,3	31 (22; 41)	10 - 48	0,009^¶^
Aún no he decidido	36,1 ±8,8	38,5 (29; 42)	13 - 50	
Sí	37,4 ±9,3	40 (31; 46)	10 - 50	
Planea estudios doctorado				
No	30,2 ±10,8	31 (23; 39)	10 - 47	<0,001^¶^
Aún no he decidido	37,9 ±9,3	40 (31; 46)	13 - 50	
Sí	36,8 ±9,0	39 (30; 43)	10 - 50	
Laborales				
Tiene trabajo remunerado				
No	37,4 ±9,0	40 (31; 46)	10 - 50	0,151^∥^
Sí	35,3 ±10,2	37 (28; 43)	10 - 50	
Sector donde trabaja (n=175)				
Trabajo en otros sectores	36,7 ±9,5	39 (30; 45)	10 - 50	0,151^∥^
Trabaja en sector salud	34,4 ±10,5	35 (26; 42)	10 - 50	
Dónde planea trabajar				
Hospital, Clínica, Instituto especializado	36,3 ±9,3	39 (30; 44)	10 - 50	<0,001^¶^
Centro o puesto de salud del PNA**	40,3 ±8,0	41 (38; 46)	13 - 50	
Instituciones dedicadas a investigación público o privado	38,6 ±8,8	43 (30; 46)	20 - 46	
Organizaciones no asistenciales vinculadas a la salud (Ministerio de Salud, OPS^††^, ONGs^‡‡^, etc)	33,5 ±10,3	34,5 (25,5; 41,5)	10 - 50	
Trabajar como profesor a nivel universitario o técnico	35,9 ±9,4	35 (27; 44)	19 - 50	
Otros	30,3 ±12,6	29 (20; 40)	10 - 50	

*IL-PNA = Intención laboral en el primer nivel de atención; ^†^DE = Desviación estándar; ^‡^RIC = Rango intercuartil; ^§^Rango = Valor mínimo y máximo; ^∥^Resultado de la prueba U de Mann-Whitney; ^¶^Resultado de la prueba H de Kruskal-Wallis; **PNA = Primer nivel de atención; ^††^OPS = Organización Panamericana de la Salud; ^‡‡^ONGs = Organizaciones no gubernamentales

En cuanto a las covariables estudiadas se encontró una tendencia negativa sobre el puntaje de IL-PNA, donde los estudiantes de sexo masculino tienen 2,51 (IC 95%: -4,63; -0,39) puntos menos en la mediana de IL-PNA respecto a las mujeres; por su parte, los estudiantes de universidades privadas tienen 2,16 (IC 95%: -3,92; -0,40) puntos menos en la mediana de IL-PNA respecto a los estudiantes de universidades públicas, mientras quienes no tienen planeado estudiar una especialización presentaban 7,48 (IC 95%: -13,81; -1,15) puntos menos en la mediana de IL-PNA respecto de quienes aún no han decidido dichos estudios. Las covariables que favorecieron el puntaje de IL-PNA fueron la preparación para el trabajo en el PNA donde quienes consideraban estar preparados (1,82; IC 95%: 0,02; 3,62) y muy preparados (4,44; IC 95%: 0,67; 8,21) presentaron una mediana mayor respecto a los que señalaban tener regular preparación; así también los estudiantes que tienen planeado estudios de especialización tuvieron 3,20 (IC 95%: 0,65; 5,75) puntos más en cuanto a la IL-PNA respecto al aún no haber tomado una decisión para dichos estudios (Tabla 5).

**Tabla 5:** Factores asociados al puntaje de IL-PNA* de estudiantes de enfermería de universidades del Perú, 2023-2024 (n = 740)

Características	Coef ajust^†^ (IC95%^‡^)	Valor de P
Puntajes PTE-PNA^§^	0,46 (0,39; 0,53)	<0,001
Puntajes C-APS^∥^	-0,02 (-0,22; 0,19)	0,866
Sociodemográficas		
Sexo		
Femenino	Referencia	
Masculino	-2,51 (-4,63; -0,39)	0,020
Estado civil		
Soltero	Referencia	
Casado	1,46 (-2,87; 5,79)	0,507
Conviviente	-0,62 (-4,42; 3,18)	0,749
Divorciado	-7,18 (-15,13; 0,77)	0,077
Otros	8,30 (-12,91; 29,5)	0,443
Tiene hijos		
No	Referencia	
Si	0,00 (-2,65; 2,64)	0,998
Dependencia económica		
No	Referencia	
Sí	0,54 (-2,00; 3,07)	0,677
Académicas		
Tipo de universidad		
Público	Referencia	
Privado	-2,16 (-3,92; -0,40)	0,016
Año de estudio		
Tercero	Referencia	
Cuarto	-0,44 (-3,07; 2,19)	0,742
Quinto	0,00 (-2,69; 2,69)	1,000
Egresado	-3,60 (-7,28; 0,09)	0,056
Pensó cambio de carrera		
No	Referencia	
Sí	-0,88 (-2,62; 0,86)	0,322
Estudios previos		
No	Referencia	
Si	-0,71 (-2,96; 1,55)	0,538
Frecuencia de prácticas en el PNA^¶^		
No hubo o fueron muy escasas prácticas	0,23 (-2,29; 2,74)	0,858
Escasas prácticas	-0,59 (-2,77; 1,59)	0,594
Prácticas moderadamente frecuentes	Referencia	
Prácticas frecuentes	-0,97 (-3,11; 1,18)	0,377
Prácticas muy frecuentes	0,40 (-2,47; 3,27)	0,785
Preparación para trabajo en el PNA^¶^		
No estoy preparada	-4,71 (-11,06; 1,63)	0,145
Algo preparada	-0,94 (-3,39; 1,50)	0,449
Regular preparada	Referencia	
Estoy preparada	1,82 (0,02; 3,62)	0,048
Estoy muy preparada	4,44 (0,67; 8,21)	0,021
Planea estudios especialización		
No	-7,48 (-13,81; -1,15)	0,021
Aún no he decidido	Referencia	
Sí	3,20 (0,65; 5,75)	0,014
Planea estudios maestría		
No	-0,55 (-6,20; 5,10)	0,848
Aún no he decidido	Referencia	
Sí	0,40 (-1,81; 2,61)	0,723
Planea estudios doctorado		
No	-2,29 (-6,61; 2,04)	0,299
Aún no he decidido	Referencia	
Sí	-0,76 (-2,55; 1,04)	0,409
Laborales		
Tiene trabajo remunerado		
No	Referencia	
Sí	0,05 (-2,57; 2,67)	0,970

*IL-PNA = Intención laboral en el primer nivel de atención; ^†^Coef Ajust = Coeficiente ajustado para regresión de cuantiles a la mediana; ^‡^IC95% = Intervalo de confianza al 95%; ^§^PTE-PNA = Percepción del trabajo de la enfermera en el primer nivel de atención; ^∥^C-APS = Conocimientos sobre atención primaria de salud; ^¶^PNA = Primer nivel de atención

## Discusión

Las enfermeras desempeñan un rol fundamental en el PNA, siendo en muchos territorios y establecimientos de salud del PNA el único profesional de la salud disponible, principalmente en zonas remotas y rurales^9^ ^,^ ^27^. Sin embargo, la escasez de enfermeras es un problema crítico con tendencia a empeorar por la potencial deserción de enfermeras del PNA y la evasión de este nivel como ámbito de desarrollo laboral. 

El estudio muestra una notoria IL-PNA en los estudiantes revelando una disposición favorable al PNA; que se sustenta en creencias normativas positivas de valoración del PNA y la AP; como la identificación de docentes, referentes y políticas de APS como motivadores; y la autopercepción de preparación para desempeñarse en el PNA. Estos hallazgos reflejan avances en el reconocimiento del PNA como ámbito de desarrollo profesional y de la APS como eje de la atención integral. Aunque la literatura reciente en Latinoamérica es escasa, los resultados concuerdan con un estudio realizado en Perú que identificó una mayor disposición de los estudiantes de enfermería para trabajar en áreas rurales que entre los estudiantes de medicina^28^. Contrariamente, estudios en México (1,9%)^18^ y Perú (12,5%)^19^ reportaron muy baja IL-PNA. 

Las percepciones del trabajo de la enfermera en el PNA fueron mayormente desfavorables, destacando valoraciones negativas sobre prestigio, condiciones laborales, rol profesional en el PNA, oportunidades de desarrollo, liderazgo y remuneración. Estos resultados son consistentes con un estudio realizado en Brasil^29^ que reveló tensiones entre el ideal de la APS (vínculo, territorio, integralidad) y las limitaciones estructurales, se reconoce la complejidad del rol de enfermería en el PNA/AP que combina actividades asistenciales, de gestión y de promoción de la salud, donde la construcción de vínculos con los usuarios, el conocimiento del territorio, trabajo con la comunidad y el enfoque intercultural, constituyen competencias centrales; y que la sobrecarga administrativa, escasez de recursos y las dificultades en el trabajo interdisciplinario, limitan el desarrollo pleno de la práctica de enfermería en este nivel^29^.

Respecto a conocimientos sobre APS predominó un nivel moderado, consistente con otro estudio en Perú^30^ en personal de salud. La evaluación analizó aspectos básicos de la APS (valores, principios, elementos) e importancia de sistemas de salud basados en APS^31^. Estos resultados demandan una integración más sólida de la APS en la formación de enfermería. Por otro lado, la relación inversa no significativa entre conocimiento e IL- PNA encontrado y la limitada evidencia latinoamericana al respecto resalta la necesidad de su exploración en otros países. Esta valoración de los conocimientos en estudiantes próximos a graduarse permitiría ajustar los planes de estudio de enfermería. En este marco, el instrumento diseñado y validado para Perú constituye una base valiosa para futuras adaptaciones y optimizaciones en otros entornos. 

El estudio evidenció que percepciones positivas del trabajo de enfermería en el PNA y la autopercepción de preparación se asocian a mayor intención laboral en el PNA, mientras que ser varón y estudiar en universidad privada influyen negativamente. Estos hallazgos ponen de manifiesto la urgencia de potenciar el proceso formativo en APS. Estudios previos entre estudiantes no se encontraron. Un estudio que incluyó a enfermeros de AP en Brasil observó que las percepciones favorables del entorno laboral se vincularon con mayor permanencia y satisfacción laboral^32^. Estos resultados sugieren que mejorar la percepción estudiantil del rol de enfermería en el PNA puede contribuir a atraerlas a este nivel, siendo esta percepción un posible predictor temprano de la elección de carrera, un aspecto que debe abordarse durante la formación. Estas percepciones son dinámicas y se moldean con experiencias, entornos institucionales, modelos a seguir e interacción con docentes y tutores, lo que plantea retos significativos para la formación^33^ ^-^ ^34^ y subraya la necesidad de reflexión dentro de las instituciones formadoras.

Ante el déficit de enfermeros en América Latina, promover la vocación hacia el PNA/AP es esencial para reducir brechas, avanzar hacia sistemas de salud equitativos y la CUS. Las universidades y escenarios de práctica resultan claves para formar estudiantes competentes y con percepciones y actitudes positivas al PNA/APS. En este sentido, apremian reformas y acciones estratégicas en las universidades para consolidar la formación en enfermería con enfoque al PNA/APS^33^ ^-^ ^34^, formando profesionales íntegros y humanistas con competencias^31^ y herramientas que les permitan interactuar con las comunidades, responder a la diversidad, abordar los determinantes sociales y satisfacer las necesidades de las comunidades.

Los países buscan consolidar sus sistemas de salud sustentados en la APS, y las universidades deben responder y contribuir a este esfuerzo. Por lo tanto, estas reformas implican rediseñar los planes de estudio con un enfoque explícito en la APS/PNA^35^, superando el modelo biomédico^34^, reconceptualizar y transformar el sentido de las prácticas de enfermería en la comunidad^36^ promover la práctica temprana y extendida en comunidades y áreas rurales, fortalecer la formación y función docente y acompañamiento de tutores^37^, innovar estrategias para la educación interprofesional, la simulación^38^ ^-^ ^39^ y la evaluación de las percepciones estudiantiles. La evidencia muestra que programas educativos con experiencias prácticas potencian competencias^34^, mejoran la valoración del rol de la enfermería en AP y estimulan el interés laboral. Sin embargo, aún persisten limitaciones en currículos con escasa presencia comunitaria y ausencia de referentes profesionales a seguir, lo que restringe la comprensión del campo de acción de la enfermería en la AP^34^.

Los hallazgos, aportan evidencia útil para orientar políticas laborales que favorezcan la atracción y retención de enfermeros en el PNA. Dado su impacto significativo en la “percepción”, es necesario mejorar las condiciones laborales desfavorables y abordar la insatisfacción percibida dentro de los centros del PNA/AP y en ámbitos comunitarios y rurales^6^ ^,^ ^9^ ^,^ ^14^ ^,^ ^29^ ^,^ ^40^ ^-^ ^41^. Las realidades difieren entre países y dentro de ellos, cada uno con peculiaridades únicas que requieren análisis. La gobernanza eficaz de los sistemas de salud es fundamental para fortalecer el PNA y la APS^42^ con mayor inversión en recursos y presupuestos para el PNA, junto con políticas que mejoren integralmente las condiciones estructurales y laborales de la enfermería. 

Por otro lado, para mejorar la “percepción” se requiere abordar la sobrecarga administrativa y asistencial^27^, garantizar infraestructura adecuada, disponibilidad de suministros, capacitación y formación de posgrado, protocolos de atención^43^, mayores márgenes de autonomía en la gestión del cuidado^44^ ^-^ ^45^ y conducción de equipos^46^. Además, se debe promover el reconocimiento social y organizacional del rol de las enfermeras^13^, y brindar apoyo psicosocial que favorezca su bienestar. En lo económico, se requiere asegurar contratos estables, remuneraciones justas^13^ ^-^ ^14^ y establecer incentivos diferenciados (escalas salariales, bonos, beneficios como vivienda, transporte, alimentación entre otros) para atraer y retener enfermeros en el PNA, especialmente en zonas rurales, amazónicas y altoandinas, a fin de garantizar continuidad y acceso al cuidado integral de salud en estas áreas.

Las percepciones negativas sobre el rol de enfermería en el PNA reflejan desvalorización y escaso reconocimiento profesional, lo que coincide con una valoración social históricamente desfavorable de la enfermería^6^ ^,^ ^9^ ^,^ ^14^ ^,^ ^40^ ^,^ ^47^. Esto exige que organizaciones de enfermería, universidades, autoridades y los propios enfermeros impulsen acciones de revalorización y mayor visibilidad^48^ del impacto de su labor, reforzando la identidad y compromiso profesional^49^. El PNA por su complejidad y relevancia en el liderazgo de programas con impacto en la salud de familias y comunidades^12^, es un espacio estratégico para posicionar y empoderar a la enfermería^50^; sin embargo, requiere mejoras sostenidas en las condiciones laborales y salariales para quienes trabajan en este nivel.

Este trabajo constituye el primero con enfoque cuantitativo realizado en estudiantes peruanos. Se recomienda desarrollar estudios longitudinales multicéntricos, incluyendo métodos cualitativos para mayor comprensión de los facilitadores y obstáculos de las preferencias de trayectoria laboral de los estudiantes en diversos contextos. Sus principales limitaciones son: a) Las universidades fueron seleccionadas por conveniencia; lo cual pudo introducir sesgo de selección y limitar su representatividad; b) El diseño fue un estudio transversal por tanto no establece relaciones causales y c) Es probable que la limitada experiencia y conocimiento de los estudiantes del rol real de las enfermeras en el PNA pudo sesgar su percepción o que hayan ajustado sus respuestas por un tema de deseabilidad social. Por tanto, los resultados deben ser interpretados con cautela tomando en cuenta estas consideraciones. 

## Conclusión

Las percepciones favorables del trabajo de enfermería en el PNA tienen impacto positivo en la intención laboral de los estudiantes de enfermería en este nivel. Mientras que, el conocimiento sobre APS no tendría influencia en la intención laboral. Sin embargo, se recomienda estudios adicionales, considerando que el análisis bivariado sugirió que a mayor conocimiento sobre APS hay una menor intención de trabajo en el PNA.

La autopercepción de estar preparado para trabajar en el PNA tiene influencia favorable sobre el interés de desarrollo laboral en este nivel, contrario a lo que ocurre con estudiantes de universidades privadas y estudiantes varones. El predominio de estudiantes con nivel moderado de conocimientos sobre APS demanda una integración más sólida de la APS en la formación de enfermería.

Los hallazgos revelan la importancia del abordaje de la percepción de la imagen y rol de enfermería en el PNA durante la formación universitaria, para forjar juicios y una comprensión adecuada de la enfermería que impulse una trayectoria laboral en el PNA, además de revertir las percepciones desfavorables encontradas y superar la alta proporción de estudiantes con nivel medio de conocimientos sobre APS. 

Por otro lado, es necesario impulsar estrategias para revalorizar, empoderar y lograr mayor visibilidad de la profesión de enfermería en el PNA, así como abogar por mejores condiciones laborales en el PNA y por el fortalecimiento de la propia APS, para revertir las circunstancias adversas que enfrenta y hacer que el trabajo en este ámbito sea más atractivo.

## Data Availability

Los conjuntos de datos relacionados con este artículo estarán disponibles previa solicitud al autor correspondiente.
